# Effects of aspirin on risks of vascular events and cancer according to bodyweight and dose: analysis of individual patient data from randomised trials

**DOI:** 10.1016/S0140-6736(18)31133-4

**Published:** 2018-08-04

**Authors:** Peter M Rothwell, Nancy R Cook, J Michael Gaziano, Jacqueline F Price, Jill F F Belch, Maria Carla Roncaglioni, Takeshi Morimoto, Ziyah Mehta

**Affiliations:** a Centre for Prevention of Stroke and Dementia, Nuffield Department of Clinical Neuroscience, University of Oxford, Oxford, UK; b Division of Preventive Medicine, Department of Medicine, Brigham and Women's Hospital, Boston, MA, USA; c Harvard Medical School, Boston, MA, USA; d Usher Institute of Population Health Sciences and Informatics, University of Edinburgh, Edinburgh, UK; e Institute of Cardiovascular Research, Vascular and Inflammatory Diseases Research Unit, University Division of Medicine and Therapeutics, Ninewells Hospital and Medical School, Dundee, UK; f Istituto di Ricerche Farmacologiche Mario Negri, Milan, Italy; g Department of Clinical Epidemiology, Hyogo College of Medicine, Nishinomiya, Japan

## Abstract

**Background:**

A one-dose-fits-all approach to use of aspirin has yielded only modest benefits in long-term prevention of cardiovascular events, possibly due to underdosing in patients of large body size and excess dosing in patients of small body size, which might also affect other outcomes.

**Methods:**

Using individual patient data, we analysed the modifying effects of bodyweight (10 kg bands) and height (10 cm bands) on the effects of low doses (≤100 mg) and higher doses (300–325 mg or ≥500 mg) of aspirin in randomised trials of aspirin in primary prevention of cardiovascular events. We stratified the findings by age, sex, and vascular risk factors, and validated them in trials of aspirin in secondary prevention of stroke. Additionally, we assessed whether any weight or height dependence was evident for the effect of aspirin on 20-year risk of colorectal cancer or any in-trial cancer.

**Results:**

Among ten eligible trials of aspirin in primary prevention (including 117 279 participants), bodyweight varied four-fold and trial median weight ranged from 60·0 kg to 81·2 kg (p<0·0001). The ability of 75–100 mg aspirin to reduce cardiovascular events decreased with increasing weight (p_interaction_=0·0072), with benefit seen in people weighing 50–69 kg (hazard ratio [HR] 0·75 [95% CI 0·65–0·85]) but not in those weighing 70 kg or more (0·95 [0·86–1·04]; 1·09 [0·93–1·29] for vascular death). Furthermore, the case fatality of a first cardiovascular event was increased by low-dose aspirin in people weighing 70 kg or more (odds ratio 1·33 [95% CI 1·08–1·64], p=0·0082). Higher doses of aspirin (≥325 mg) had the opposite interaction with bodyweight (difference p_interaction_=0·0013), reducing cardiovascular events only at higher weight (p_interaction_=0·017). Findings were similar in men and women, in people with diabetes, in trials of aspirin in secondary prevention, and in relation to height (p_interaction_=0·0025 for cardiovascular events). Aspirin-mediated reductions in long-term risk of colorectal cancer were also weight dependent (p_interaction_=0·038). Stratification by body size also revealed harms due to excess dosing: risk of sudden death was increased by aspirin in people at low weight for dose (p_interaction_=0·0018) and risk of all-cause death was increased in people weighing less than 50 kg who were receiving 75–100 mg aspirin (HR 1·52 [95% CI 1·04–2·21], p=0·031). In participants aged 70 years or older, the 3-year risk of cancer was also increased by aspirin (1·20 [1·03–1·47], p=0·02), particularly in those weighing less than 70 kg (1·31 [1·07–1·61], p=0·009) and consequently in women (1·44 [1·11–1·87], p=0·0069).

**Interpretation:**

Low doses of aspirin (75–100 mg) were only effective in preventing vascular events in patients weighing less than 70 kg, and had no benefit in the 80% of men and nearly 50% of all women weighing 70 kg or more. By contrast, higher doses of aspirin were only effective in patients weighing 70 kg or more. Given that aspirin's effects on other outcomes, including cancer, also showed interactions with body size, a one-dose-fits-all approach to aspirin is unlikely to be optimal, and a more tailored strategy is required.

**Funding:**

Wellcome Trust and National Institute for Health Research Oxford Biomedical Research Centre.

## Introduction

Aspirin inhibits platelet aggregation by irreversible acetylation of the cyclo-oxygenase-1 (COX-1) enzyme, resulting in almost complete inhibition of thromboxane production by platelets.^1^ However, aspirin yields only modest long-term reductions in vascular events,^2^, ^3^ which has led investigators to develop alternative antiplatelet drugs and to study the effects of their combination with aspirin and of dual treatment with anticoagulant drugs. Yet the disparity between the effect of aspirin on thromboxane production and its clinical benefits might be due, at least in part, to the one-dose-fits-all approach used in trials and clinical practice, particularly the use of low doses in individuals with higher bodyweight. Obesity and increased body-mass index (BMI) are associated with reduced inhibition of COX-1 by low doses of aspirin, probably due to increased platelet activation or turnover,^4^, ^5^ but high lean body mass could also reduce the systemic bioavailability of aspirin. Aspirin is rapidly de-acetylated by esterases in the intestinal wall, plasma, red blood cells, and liver,^6^ and so the proportion of a fixed dose that reaches the systemic circulation will depend on the mass of these tissues, which is correlated with lean body size. Given that aspirin acetylates several highly abundant proteins such as albumin, haemoglobin, and fibrinogen, their masses might also affect the systemic bioavailability of aspirin. Although about 50% of an oral dose of aspirin reaches the portal circulation^6^—and can therefore inhibit circulating platelets—reduced systemic bioavailability could restrict inhibition of COX-1 in megakariocytes and, hence, in the 10–15% of new platelets that are released daily.^7^ Thus, reduced systemic bioavailability of once-daily, low-dose aspirin at higher lean body mass could reduce clinical effectiveness, especially if doses are missed. Total bodyweight could be a particularly powerful determinant of clinical effects if obesity also increases platelet turnover.

**Research in context**

**Evidence before this study**

A one-dose-fits-all approach to use of aspirin has yielded only modest benefits in the long-term prevention of cardiovascular events, possibly due to underdosing in patients of high body size and excess dosing in those of low body size. Randomised trials of aspirin in primary prevention of cardiovascular events have all tested a single dose against a control, but have differed in the dose(s) chosen. We identified ten randomised trials of aspirin in primary prevention (involving 117 279 participants) from the Antithrombotic Trialists' Collaboration, the Cochrane Collaboration Database of Systematic Reviews, and previous systematic reviews.

**Added value of this study**

In the absence of previous analyses, we used individual patient data from trials of aspirin versus control in the primary prevention of cardiovascular events, stratifying by weight, height, and other measures of body size, to identify whether the effects of high and low doses of aspirin are modified by these variables. We validated findings in trials of aspirin in secondary prevention of stroke, and by determining any weight dependence of the effects of aspirin on long-term risk of colorectal cancer and on short-term risk of all cancer. We found that the ability of low-dose aspirin (75–100 mg) to reduce cardiovascular events declined with increasing weight, with substantial benefit at 50–69 kg but no benefit at 70 kg or more, and with increased case fatality of first cardiovascular events in people weighing 70 kg or more. Higher doses (≥325 mg) of aspirin showed a reverse interaction with weight and height, reducing cardiovascular events only at larger body size. Findings were consistent in men and women, in participants with diabetes, and in trials of aspirin in secondary prevention of stroke. Reductions in long-term risk of colorectal cancer by aspirin were both height and weight dependent. However, stratification by body size revealed harms due to excess dosing, with an increase in sudden deaths at lower weight and an increase in the short-term risk of cancer at lower weight and shorter height in participants aged 70 years or older.

**Implications of all the available evidence**

The optimal dose of aspirin to prevent cardiovascular events depends on bodyweight, driven more by lean body mass and height than by body-mass index. Once-daily low doses (75–100 mg) of aspirin were ineffective in people weighing 70 kg or more, particularly in those who smoke or were treated with enteric-coated formulations, whereas higher doses became more effective with increasing weight. We also found that the effects of aspirin on sudden cardiac death and cancer showed dose–weight interactions, suggesting that the one-dose-fits-all strategy for daily aspirin is unlikely to be optimal. The substantial reductions in cardiovascular events and death at optimal doses for weight highlight the potential to improve effectiveness and argue for a more tailored dosing strategy.

Higher doses of aspirin should overcome any reduced bioavailability with increasing body size, but might be excessive in patients with low bodyweight because of reduced endothelial prostacyclin production due to high systemic levels of aspirin^8^ or possibly because of increased salicylate levels. If the effectiveness of lower doses decreases, and the effectiveness of higher doses increases, with increasing body size, then weight–dose interactions could explain why low-dose aspirin appears to prevent stroke only in women,^9^ and high doses only in men,^10^ despite them having similar BMIs.

All randomised trials of aspirin in prevention of vascular events have tested a one-dose-fits-all approach (ie, one dose *vs* placebo or, occasionally, comparing two different doses applied to all), but have differed in the doses chosen. Previous analyses of aspirin trials have not found consistent effect modification for BMI,^3^, ^11^ but trials of different doses have been analysed together, and BMI is poorly correlated with lean body size. In the absence of previous analyses, we aimed to investigate the modifying effects of weight, height, BMI, and other measures of body size on the effectiveness of low and higher doses of aspirin in primary prevention of vascular events, with validation in trials in secondary prevention of stroke. Given that aspirin also has effects on risk of cancer,^12^, ^13^ which might partly depend on platelet inhibition,^1^ we also investigated whether the effect of aspirin on long-term risk of colorectal cancer, and on short-term, in-trial risk of any cancer, was affected by weight and height.

## Methods

### Search strategy and selection criteria

Trials of aspirin versus control in primary prevention of vascular events were identified from the Antithrombotic Trialists' (ATT) Collaboration,^3^, ^11^ from other previous systematic reviews of trials of aspirin, and from the Cochrane Collaboration Database of Systematic Reviews. Trials were eligible if they randomly assigned participants to daily or alternate-day aspirin versus no aspirin. Trials that included factorial randomisation to other interventions were also eligible. Trials of aspirin versus control in secondary prevention of stroke that randomised at least 1000 participants were identified from a previous systematic review,^14^ along with any trials comparing different doses of aspirin in the secondary prevention of stroke identified by the ATT.^3^, ^11^ Trials of short-term (≤90 days) treatment were excluded.

For all eligible trials, we obtained individual patient data on age, sex, weight, height, and vascular risk factors (including smoking status and diabetes status) at baseline, and on all major vascular events (including stroke [ischaemic, intracerebral, or subarachnoid haemorrhage], myocardial infarction, vascular death, other coronary death, and other major ischaemic vascular events, excluding unstable angina and transient ischaemic attack), major bleeds (intracerebral haemorrhage and extracranial bleeds that were fatal or required blood transfusion or hospital admission), cancers (first cancer, excluding non-melanoma skin cancer, diagnosed after randomisation), and deaths that occurred during follow-up. When possible, ATT Collaboration definitions of events were used.^3^, ^11^ When data were available, recurrences of pre-trial cancers were excluded, but all deaths due to cancer were included in analyses of that outcome. The designation of cancer death from the original trials was used.

Data were also obtained if trials had coded sudden deaths (ie, collapse or unwitnessed death with no known non-cardiac cause and no clinical or autopsy evidence of fatal myocardial infarction). Dates of randomisation, of all events, of withdrawal from randomised treatment, and of final follow-up were also obtained. Data on 20-year risk of colorectal cancer were obtained from trials of aspirin in primary prevention of vascular events that did post-trial follow-up.^15^, ^16^, ^17^, ^18^, ^19^, ^20^ Methods of post-trial follow-up for cancer have been reported previously for four trials,^12^, ^13^, ^21^, ^22^ and methods used previously in the UK trials^12^, ^13^ were applied in two further trials.^19^, ^20^

### Statistical analysis

Trials were analysed separately according to whether they were investigating aspirin for primary or secondary prevention and whether they used low doses (≤100 mg) or higher doses (≥300 mg) of aspirin. Primary and secondary prevention trials were pooled only if the effect modification by weight or height was similar in both settings. Analyses of the effects of aspirin were done by intention to treat based on randomised allocation, unless otherwise specified. For analyses stratified by baseline characteristics, data on age and sex were complete, data on smoking were 99·9% complete, and data on weight or height were missing in about 0·1% of participants (range 0–1·05; appendix pp 2, 3); thus, participants with missing data were simply excluded.

Participants in each trial were first dichotomised by bodyweight: those weighing less than 70 kg versus those weighing 70 kg or more. For each outcome, hazard ratios (HRs) were calculated for aspirin versus control in each trial, pooled estimates were obtained by fixed-effects meta-analysis (Mantel-Haenszel-Peto method), and heterogeneity was calculated with the χ^2^ test. In the absence of significant (p<0·05) heterogeneity in estimates between trials, individual patient data were pooled and the effects of aspirin in people weighing less than 70 kg versus those weighing 70 kg or more were estimated with a Cox model stratified by trial. Kaplan-Meier curves were also generated for time to event and dichotomised by bodyweight (<70 kg *vs* ≥70 kg), with significance established by use of the log-rank test stratified by trial. Participants without an event were censored on date of death or at the end of trial follow-up. This analysis was repeated with censoring at time of discontinuation of randomised treatment.

Given that aspirin can affect the severity of vascular events,^14^ we also did weight-stratified analyses of the effects of aspirin on fatal events only, and on case fatality of events for stroke, myocardial infarction, and all cardiovascular events during trial follow-up. Additionally, we determined the effects of aspirin on risk of all cardiovascular events or death from any cause. Because effects on fatal events in trials can be diluted by active treatment after non-fatal events,^23^ these analyses were limited to death and case fatality due to first events during follow-up and were also repeated with censoring at discontinuation of randomised treatment.

Any interaction between the effect of aspirin on outcome and bodyweight was assessed by including in the Cox model an interaction term between weight and treatment for the analysis of participants weighing less than 70 kg versus those weighing 70 kg or more and with additional analyses with the interaction term based on weight as a continuous variable. Effects of aspirin were also determined in 10 kg bands of weight (<50, 50–59, 60–69, 70–79, 80–89, and ≥90), and in 0·1 m bands of height (<1·40, 1·40–1·49, 1·50–1·59, 1·60–1·69, 1·70–1·79, and ≥1·80), which were shown graphically as HRs (95% CIs). When height had been recorded in feet and inches, corresponding thresholds in metres were estimated. Effects of aspirin were also compared in tall (top quintile within each sex) versus shorter (lower four quintiles) individuals. Analyses stratified by weight were also repeated after exclusion of participants who were underweight (BMI <18·5 kg/m^2^) or who were obese (BMI ≥30 kg/m^2^). Effect modification by other measures of body size (including lean body mass, BMI, fat mass, and body surface area; definitions are in appendix p 13) and by vascular risk factors was also assessed by use of interaction terms in Cox models.

Analysis of the effect of aspirin on risk of all cardiovascular events stratified by weight was further stratified by age (<70 years *vs* ≥70 years), sex, smoking status (current smoker *vs* previous or never smoker), BMI (<25 kg/m^2^ *vs* ≥25 kg/m^2^), formulation of aspirin tablet used (enteric coated or delayed release *vs* standard release), period of follow-up (<3 years *vs* ≥3 years), and whether or not the participant was also randomised to vitamin E in trials with this factorial design.

For the effect of aspirin on risk of all cardiovascular events, the significance of any differences in effect modification by weight and height between low-dose (≤100 mg) and higher-dose (≥300 mg) aspirin was established with the Mantel-Haenszel-Peto heterogeneity statistic for difference between low-dose versus higher-dose interaction estimates derived from a Cox model for weight–treatment or height–treatment interactions.

In the primary prevention trials with post-trial follow-up for cancer, the effect of aspirin on the 20-year risk of colorectal cancer was stratified by weight in the same way as described for vascular events, with additional stratification by age (<70 years *vs* ≥70 years) and dose of aspirin (75–100 mg *vs* ≥300 mg). In all primary prevention trials, the same analysis was done for risk of first cancer during trial follow-up, with additional stratification according to sex, diabetes status, and period of follow-up. In line with previous analyses,^12^, ^13^, ^14^ we selected follow-up periods of less than 3 years, 3–4·9 years, and 5 years or more.

### Role of the funding source

The funders of the study had no role in study design, data collection, data analysis, data interpretation, or writing of the report. The corresponding author had full access to all the data in the study and had final responsibility for the decision to submit for publication.

## Results

### Trial inclusion

We identified ten eligible trials^15^, ^16^, ^17^, ^18^, ^19^, ^20^, ^24^, ^25^, ^26^, ^27^ of aspirin versus control in primary prevention of cardiovascular events. All but one trial^20^ had collected data on weight or height, and individual patient data on baseline characteristics and all major cardiovascular events were available from nine trials (appendix pp 2, 3).^15^, ^16^, ^17^, ^18^, ^19^, ^20^, ^24^, ^25^, ^26^ Seven trials^17^, ^18^, ^19^, ^20^, ^24^, ^25^, ^26^ investigated low doses of aspirin (75–100 mg) versus control and two trials^15^, ^16^ investigated higher doses of aspirin versus control. We also identified five eligible trials^28^, ^29^, ^30^, ^31^, ^32^ of aspirin in secondary prevention of stroke and obtained individual patient data from the four largest trials: one of low-dose aspirin versus placebo,^28^ two of higher doses of aspirin versus placebo,^29^, ^30^ and one comparing two doses (appendix p 3).^31^ The only other eligible trial^32^ of low-dose aspirin in secondary prevention of stroke did not collect data on weight or height.

Bodyweight varied approximately four-fold in each of the trials (appendix pp 2, 3). Median weight ranged from 60·0 kg to 81·2 kg (p<0·0001), in part due to sex differences (median weight was 81·0 kg in men and 68·0 kg in women). Trials also differed in age of participants and in number of participants who smoked.

### Low-dose aspirin

In the initial meta-analysis of trials of low-dose aspirin in primary prevention, pooled odds ratios (ORs) for the effect of aspirin on risk of cardiovascular events were 0·77 (95% CI 0·68–0·87, p<0·0001; p_heterogeneity_=0·32) for participants weighing less than 70 kg versus 0·94 (0·86–1·04, p=0·24; p_heterogeneity_=0·50) for those weighing 70 kg or more (table 1). Given the lack of heterogeneity in these effects between studies, we proceeded to do the pooled analyses.

**Table 1:** Pooled analysis of the effect of low-dose aspirin versus control in primary prevention of vascular events according to bodyweight, age, sex, presence of diabetes, and current smoking

	**Bodyweight <70 kg**				**Bodyweight ≥70 kg**				**All**			
	Aspirin	Control	HR (95% CI)	p value	Aspirin	Control	HR (95% CI)	p value	Aspirin	Control	HR (95% CI)	p value
**Stroke**												
All	198	279	0·71 (0·59–0·85)	0·0002	302	302	1·00 (0·86–1·18)	0·97	500	581	0·86 (0·76–0·97)	0·014
Women	161	218	0·74 (0·60–0·90)	..	147	172	0·86 (0·69–1·08)	..	308	390	0·79 (0·68–0·92)	*..*
Men	37	61	0·62 (0·41–0·93)	..	155	130	1·18 (0·94–1·49)	*..*	192	191	1·00 (0·82–1·22)	..
With diabetes	45	73	0·60 (0·41–0·87)	..	63	75	0·84 (0·60–1·18)	*..*	108	148	0·72 (0·56–0·93)	..
Smokers	59	77	0·74 (0·53–1·04)	..	86	56	1·57 (1·12–2·19)	*..*	145	133	1·09 (0·86–1·37)	..
Age ≥70 years	64	83	0·70 (0·51–0·97)	..	54	60	0·91 (0·63–1·32)	*..*	118	143	0·79 (0·62–1·01)	..
**Myocardial infarction**												
All	165	199	0·81 (0·66–1·00)	0·046	381	426	0·90 (0·78–1·03)	0·14	546	625	0·87 (0·78–0·98)	0·019
Women	120	131	0·91 (0·71–1·16)	..	152	143	1·08 (0·86–1·36)	*..*	272	274	1·00 (0·84–1·18)	..
Men	45	68	0·64 (0·44–0·93)	..	229	283	0·81 (0·68–0·96)	*..*	274	351	0·77 (0·66–0·90)	..
With diabetes	35	47	0·70 (0·45–1·09)	..	114	97	1·21 (0·92–1·58)	*..*	149	144	1·03 (0·82–1·30)	..
Smokers	68	63	1·05 (0·75–1·48)	..	145	138	1·06 (0·84–1·34)	*..*	213	201	1·06 (0·87–1·28)	..
Age ≥70 years	35	46	0·64 (0·41–1·00)	..	43	37	1·25 (0·80–1·94)	*..*	78	83	0·90 (0·66–1·23)	..
**Vascular death**												
All	128	160	0·79 (0·63–1·00)	0·048	296	274	1·09 (0·93–1·29)	0·30	424	434	0·98 (0·86–1·12)	0·76
Women	90	114	0·78 (0·59–1·03)	..	107	100	1·09 (0·83–1·43)	..	197	214	0·92 (0·76–1·12)	..
Men	38	46	0·85 (0·55–1·30)	..	189	174	1·09 (0·88–1·33)	*..*	227	220	1·03 (0·86–1·24)	..
With diabetes	26	42	0·59 (0·36–0·97)	..	76	68	1·14 (0·82–1·58)	*..*	102	110	0·92 (0·71–1·21)	..
Smokers	41	51	0·78 (0·52–1·18)	..	89	88	1·02 (0·76–1·38)	*..*	130	139	0·93 (0·74–1·19)	..
Age ≥70 years	51	67	0·67 (0·47–0·97)	..	72	54	1·38 (0·97–1·97)	*..*	123	121	0·99 (0·77–1·27)	..
**All cardiovascular events**												
All	419	537	0·77 (0·68–0·87)	<0·0001	791	840	0·95 (0·86–1·04)	0·24	1210	1377	0·88 (0·81–0·95)	0·0008
Women	322	400	0·80 (0·69–0·92)	..	346	362	0·97 (0·83–1·12)	..	668	762	0·88 (0·79–0·97)	..
Men	97	137	0·70 (0·54–0·91)	..	445	478	0·92 (0·81–1·05)	..	542	615	0·87 (0·77–0·98)	..
With diabetes	91	125	0·69 (0·53–0·91)	..	196	183	1·08 (0·88–1·32)	..	287	308	0·92 (0·78–1·08)	..
Smokers	143	158	0·88 (0·70–1·10)	..	259	222	1·19 (0·99–1·42)	..	402	380	1·05 (0·92–1·21)	..
Age ≥70 years	125	159	0·70 (0·55–0·88)	..	135	119	1·16 (0·91–1·49)	..	260	278	0·90 (0·76–1·07)	..

HR=hazard ratio.

**Figure 1:**
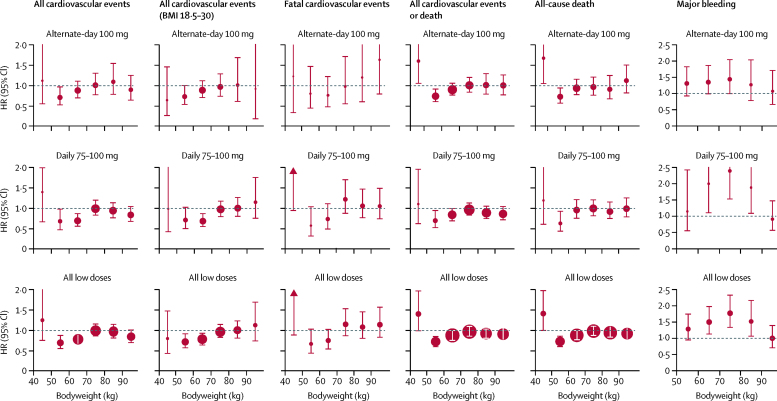
Effect of low-dose aspirin versus control on risks of cardiovascular events, death, and major bleeding according to bodyweight in trials of aspirin in primary prevention The size of the circles representing the point estimates of the HRs is proportional to the inverse of the variance of the estimate. BMI=body-mass index. HR=hazard ratio.

In the pooled analysis of trials of low-dose aspirin in primary prevention, the ability of 75–100 mg aspirin to reduce cardiovascular events decreased with increasing weight (p_interaction_=0·0072; table 1, figure 1). Low-dose aspirin had the greatest effect on cardiovascular events in participants weighing 50–69 kg (383 events in 15 155 participants treated with aspirin *vs* 504 events in 15 145 participants treated with control; HR 0·75 [95% CI 0·65–0·85]; p<0·0001), particularly with daily use (172 of 4432 treated with aspirin *vs* 245 of 4400 treated with control; 0·68 [0·56–0·83], p=0·0001; figure 1). In the one trial^18^ of alternate-day dosing, 100 mg aspirin was effective in participants weighing 50–59 kg (72 of 4325 *vs* 102 of 4408; 0·72 [0·52–0·96], p=0·025; figure 1) and in those weighing 60–69 kg who were not allocated to vitamin E (appendix p 4). However, the reduction in cardiovascular events with 75–100 mg aspirin in people weighing 50–59 kg was not seen in people weighing less than 50 kg (1·25 [0·74–2·09], p=0·40; figure 1), who also had an increased risk of all-cause death (1·52 [1·04–2·21], p=0·031). No hazard was evident in people weighing less than 50 kg after exclusion of people with a BMI of less than 18·5 kg/m^2^ (19 of 724 *vs* 22 of 696; 0·80 [0·43–1·47], p=0·47; figure 1).

Low-dose aspirin (75–100 mg) prevented stroke in women but not in men (table 1; p_interaction_=0·020), but no difference remained after accounting for weight (p_interaction_=0·20; table 1; appendix p 5). The effect of low-dose aspirin on cardiovascular events was not modified by the presence of diabetes (weight-adjusted p_interaction_=0·47) or by age (weight-adjusted p_interaction_=0·94; table 1; appendix p 5) The weight dependence of the effect was similar for on-treatment analyses (intention to treat *vs* censoring at treatment discontinuation), for early follow-up (0–3 years), and for later (≥3 years) follow-up (appendix pp 6, 7). However, the effect of low-dose aspirin on cardiovascular events was reduced in smokers (p_interaction_=0·0026; table 1; appendix p 5). Weight remained a significant effect modifier after inclusion of age, sex, and smoking interactions in the model (p_interaction_=0·0035). The effects of weight and smoking were additive overall (table 2), and were generally consistent within individual trials (appendix p 8), with possible harm from aspirin in participants who smoked and weighed 70 kg or more (table 2). The dual interaction with weight and smoking also explained why low-dose aspirin prevented myocardial infarction in men (HR 0·77 [95% CI 0·66–0·90], p=0·0014) but not in women (1·00 [0·84–1·18], p=0·96; p_interaction_=0·031), even when stratified by weight (table 1). The risk of myocardial infarction was only reduced in women who did not smoke and weighed less than 70 kg (HR 0·71 [95% CI 0·52–0·97, p=0·031] *vs* 1·33 [0·85–2·09, p=0·21] for women who smoked and weighed ≥70 kg).

**Table 2:** Effect of low-dose aspirin versus control on risk of cardiovascular events according to weight and smoking status

			**Aspirin**	**Control**	**HR (95% CI)**	**p_interaction_**
All cardiovascular events						
	All participants		..	..	..	<0·0001
		Neither	275	377	0·73 (0·62–0·85)	..
		Either	675	775	0·86 (0·78–0·96)	..
		Both	259	222	1·18 (0·99–1·41)	..
	With diabetes		..	..	..	0·0009
		Neither	64	92	0·68 (0·49–0·93)	..
		Either	159	177	0·89 (0·72–1·11)	..
		Both	64	39	1·58 (1·06–2·35)	..
	Without diabetes		..	..	..	0·0039
		Neither	211	285	0·74 (0·62–0·88)	..
		Either	516	598	0·85 (0·76–0·96)	..
		Both	195	183	1·10 (0·90–1·34)	..
	Age <70 years		..	..	..	0·0004
		Neither	170	237	0·72 (0·59–0·88)	..
		Either	540	652	0·82 (0·73–0·92)	..
		Both	240	207	1·18 (0·98–1·42)	..
	Age ≥70 years		..	..	..	0·0045
		Neither	105	140	0·67 (0·52–0·86)	..
		Either	135	123	1·14 (0·89–1·46)	..
		Both	19	15	1·14 (0·58–2·25)	..
	Alternate-day dose		..	..	..	0·0007
		Neither	142	197	0·72 (0·58–0·89)	..
		Either	257	267	0·96 (0·81–1·14)	..
		Both	72	53	1·40 (0·98–2·00)	..
	Daily dose		..	..	..	0·0051
		Neither	133	180	0·74 (0·59–0·92)	..
		Either	418	508	0·82 (0·72–0·93)	..
		Both	187	169	1·12 (0·91–1·37)	..
	Women		..	..	..	0·0002
		Neither	218	302	0·72 (0·61–0·86)	..
		Either	361	390	0·93 (0·80–1·07)	..
		Both	88	67	1·40 (1·02–1·92)	..
	Men		..	..	..	0·017
		Neither	57	75	0·76 (0·54–1·08)	..
		Either	314	385	0·80 (0·69–0·93)	..
		Both	171	155	1·09 (0·88–1·35)	..
All stroke			..	..	..	0·0002
	Neither		138	201	0·69 (0·56–0·86)	..
	Either		275	323	0·85 (0·72–0·99)	..
	Both		86	56	1·55 (1·11–2·17)	..
Myocardial infarction			..	..	..	0·025
	Neither		97	135	0·71 (0·55–0·92)	..
	Either		304	351	0·87 (0·74–1·01)	..
	Both		145	138	1·06 (0·84–1·33)	..
Cardiovascular-related death			..	..	..	0·20
	Neither		87	109	0·79 (0·60–1·05)	..
	Either		248	236	1·05 (0·88–1·26)	..
	Both		89	88	1·02 (0·76–1·37)	..

Neither refers to participants who weighed <70 kg and did not smoke, either refers to participants who weighed ≥70 kg or smoked, and both refers to participants who weighed ≥70 kg and smoked. Numbers of events are four fewer than listed in table 1 because of missing data on smoking status for four individuals. HR=hazard ratio.

The weight dependence of the effect of low-dose aspirin on cardiovascular events was observed for all tablet formulations (appendix pp 8,9), but loss of effect in participants weighing 70 kg or more was more evident for enteric-coated or delayed-release aspirin and alternate-day, standard-release aspirin than for daily, standard-release aspirin.

In participants weighing 70 kg or more, low-dose aspirin (75–100 mg) was associated with an increase in case fatality of first cardiovascular events (OR 1·33 [95% CI 1·08–1·64], p=0·0082; appendix p 10), particularly for myocardial infarction (1·73 [1·20–2·49], p=0·0035), with an overall increase in fatal first cardiovascular events in participants aged 70 years or older (HR 1·45 [95% CI 1·01–2·10], p=0·04).

The increased risk of major bleeding on low-dose aspirin versus control was lost in participants weighing 90 kg or more (p_interaction_=0·024; figure 1; appendix p 11).

Modification by weight of the effect of low-dose aspirin on cardiovascular events remained when stratifying by BMI (appendix p 5), and the effect was also modified by height (appendix p 12). Effect modification was similar for body surface area, lean body mass, and fat mass, but bodyweight was most informative (appendix pp 13, 14). When modelled together, effect modification by weight (p_interaction_=0·0026) and smoking (p_interaction_=0·0027) exceeded that by BMI (p_interaction_=0·30).

**Figure 2:**
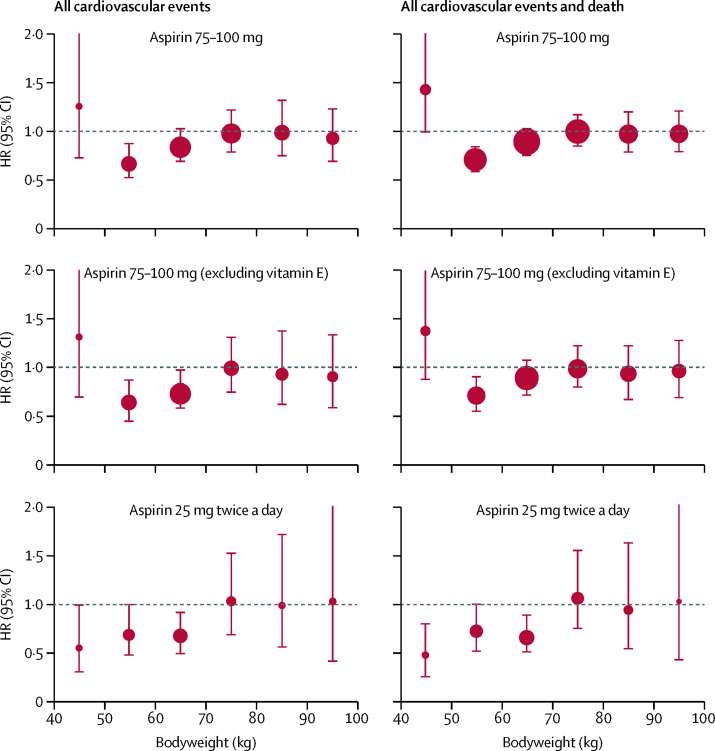
Effect of low-dose aspirin versus control on risk of all cardiovascular events and of all cardiovascular events and death in women, stratified by bodyweight The analysis of the six trials of aspirin in primary prevention is shown for all participants (top) and with exclusion of participants who were assigned to vitamin E (middle). The bottom two panels are for aspirin in secondary prevention in the ESPS-2 trial.^28^ The size of the circles representing the point estimates of the HRs is proportional to the inverse of the variance of the estimate. HR=hazard ratio.

In secondary prevention of stroke in the ESPS-2 trial,^28^ aspirin (25 mg twice daily *vs* placebo) reduced cardiovascular events in participants weighing less than 70 kg (HR 0·74 [95% CI 0·63–0·87], p=0·0003; appendix p 15). Some benefit was also evident in participants weighing 70 kg or more, but only in the acute phase of treatment (appendix p 15). Effect modification by weight during all follow-up of that trial was similar to that in the primary prevention trials of 75–100 mg aspirin, particularly in women (HR for cardiovascular events or death was 0·68 [95% CI 0·56–0·83, p=0·0001] in women weighing <70 kg and 1·02 [0·77–1·35, p=0·90] in those weighing ≥70 kg; p_interaction_=0·022; figure 2; appendix p 15). However, benefit was also seen in women who weighed less than 50 kg (HR 0·50 [95% CI 0·29–0·84, p=0·0094] for all cardiovascular events or death; appendix p 15). In the Dutch-TIA trial (aspirin 30 mg *vs* 283 mg),^31^ 30 mg aspirin also tended to be more effective than 283 mg in women weighing less than 70 kg (23 of 253 women treated with 30 mg had a cardiovascular event *vs* 47 of 319 women treated with 283 mg), although the difference was not significant (HR 0·64 [95% CI 0·39–1·06], p=0·081).

### Higher-dose aspirin

**Figure 3:**
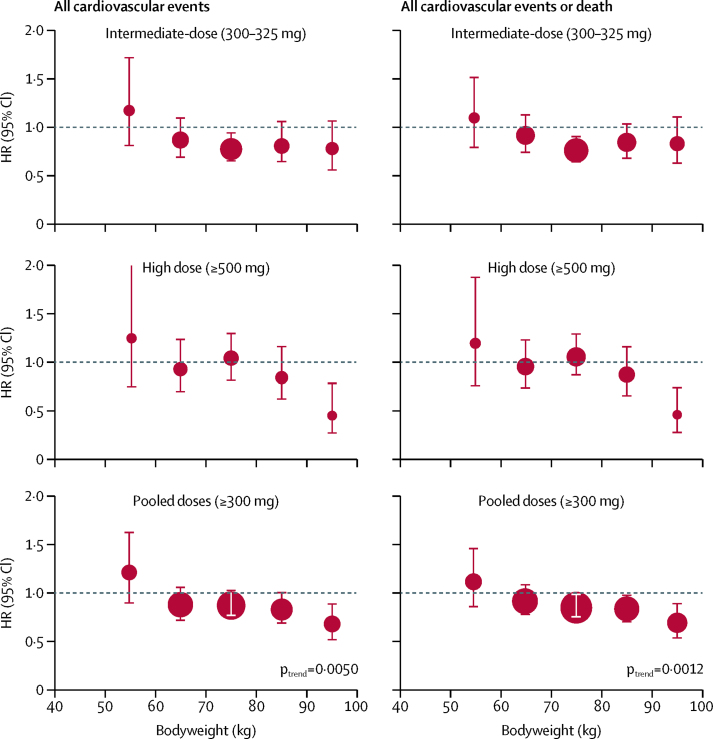
Effect of higher-dose aspirin versus control, stratified by bodyweight and dose of aspirin The size of the circles representing the point estimates of the HRs is proportional to the inverse of the variance of the estimate. The analysis included all participants in trials of higher-dose aspirin versus control in primary or secondary prevention of cardiovascular events and cardiovascular events or death (analyses confined to trials in primary prevention are in appendix pp 16, 17). HR=hazard ratio.

The ability of higher doses of aspirin (300–325 mg or 500 mg) to reduce the risk of cardiovascular events in primary prevention trials increased with weight (p_interaction_=0·017), with consistent findings for cardiovascular events or death (p_interaction_=0·0087) and in primary and secondary prevention trials combined (p_interaction_=0·0050; figure 3; appendix pp 16, 17). In primary prevention, 325 mg aspirin reduced cardiovascular events in participants weighing 70 kg or more (HR 0·83 [95% CI 0·70–0·98], p=0·028) and 500 mg aspirin reduced cardiovascular events (0·55 [0·28–1·09], p=0·086) and cardiovascular events or death (0·52 [0·30–0·89], p=0·017) in participants weighing 90 kg or more (appendix p 15). These results were consistent in a pooled analysis of all trials: cardiovascular events were reduced in participants weighing 70 kg or more (0·79 [0·70–0·90], p=0·0005) with 300–325 mg aspirin and in those weighing 90 kg or more (0·45 [0·26–0·79], p=0·0050) with 500 mg or more. The excess of major bleeding on aspirin tended to increase with weight (p_interaction_=0·087; appendix p 18).

**Figure 4:**
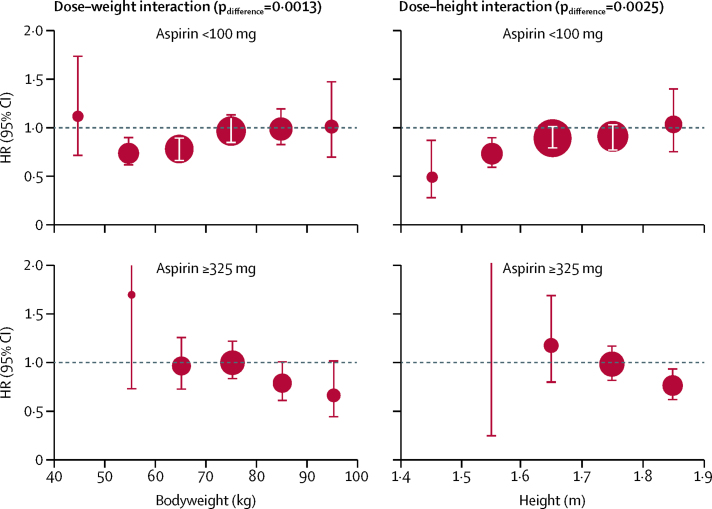
Effect of aspirin versus control on risk of cardiovascular events in trials in primary prevention, stratified by bodyweight, height, and dose The size of the circles representing the point estimates of the HRs is proportional to the inverse of the variance of the estimate. The analysis was limited to participants who were not obese (body-mass index <30 kg/m^2^). HR=hazard ratio.

The direction of the effect modification by weight for higher doses of aspirin differed from that for lower doses (difference p_interaction_=0·0013 for cardiovascular events in primary prevention trials; figure 4), and a data-derived schedule for more optimal, weight-dependent dosing (75–100 mg for people weighing 50–69 kg, 300–325 mg for those weighing 70–89 kg, and ≥500 mg for those weighing ≥90 kg) suggested that primary prevention might be improved for cardiovascular events (p_interaction_=0·0005), stroke (p_interaction_=0·0031), cardiovascular-related death (p_interaction_=0·0014), and all-cause death (p_interaction_=0·0019; appendix p 19). The direction of the effect modification by height also differed for higher doses versus lower doses of aspirin (difference p_interaction_=0·0025 for cardiovascular events in primary prevention trials; figure 4), particularly in the tallest quintile (appendix p 12).

Risk of sudden cardiac death in trials of aspirin in primary prevention was increased when the dose of aspirin exceeded that in the data-derived schedule for weight-dependent dosing (HRs were 2·03 [95% CI 1·31–3·15, p=0·0015] for all doses, 2·13 [0·88–5·19] for 75–100 mg at <50 kg, 1·99 [0·80–4·93] for 325 mg at <70 kg, and 2·26 [1·18–4·34] for 500 mg at <90 kg). By contrast, risk of sudden cardiac death was not increased at weight above those thresholds (HR 0·96 [95% CI 0·80–1·14], p=0·62; p_interaction_=0·0018; appendix p 12).

### Effects on cancer risk

**Figure 5:**
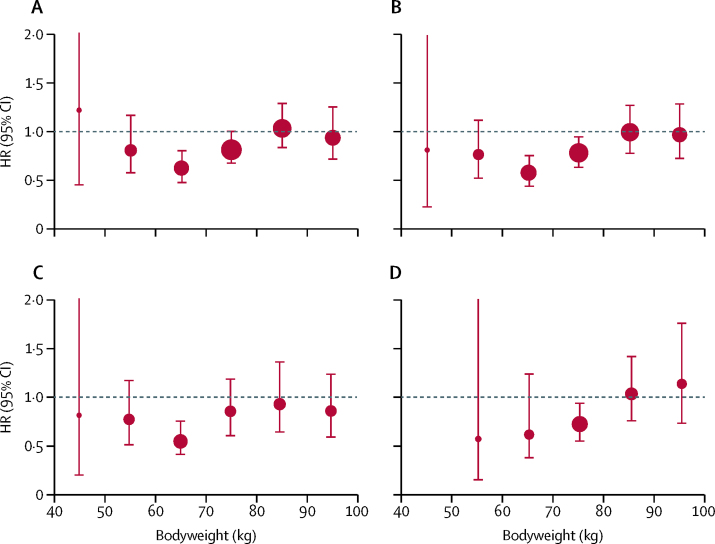
Effect of aspirin versus control on 20-year risk of colorectal cancer, stratified by aspirin dose and age The size of the circles representing the point estimates of the HRs is proportional to the inverse of the variance of the estimate. The analysis included the five trials^15^, ^16^, ^17^, ^18^, ^19^ with post-trial follow-up data and data on bodyweight. (A) Included participants treated with any dose and of any age. (B) Included participants treated with any dose who were younger than 70 years. (C) Included participants treated with 75–100 mg aspirin who were younger than 70 years. (D) Included participants treated with ≥325 mg aspirin who were younger than 70 years. HR=hazard ratio.

In the five primary prevention trials^15^, ^16^, ^17^, ^18^, ^19^ with post-trial follow-up and data on weight (including 73 372 participants), the reduction in 20-year risk of colorectal cancer (1217 cases) with aspirin was lost at higher bodyweight (p_interaction_=0·038; figure 5). Low-dose aspirin (75–100 mg) reduced risk of colorectal cancer in participants weighing less than 70 kg (HR 0·64 [95% CI 0·50–0·82], p=0·0004) but not in people weighing 70 kg or more (0·87 [0·71–1·07], p=0·32). By contrast, benefit from higher doses of aspirin (≥325 mg) extended to participants weighing up to 80 kg (0·69 [0·55–0·87, p=0·0014] for participants weighing <80 kg *vs* 1·08 [0·83–1·39] for participants weighing ≥80 kg).

Data on in-trial occurrence of cancer were available from all ten eligible trials of aspirin versus placebo in primary prevention.^15^, ^16^, ^17^, ^18^, ^19^, ^20^, ^24^, ^25^, ^26^, ^27^ Aspirin had no effect on overall number of cancer-related deaths (986 treated with aspirin *vs* 1039 treated with control; HR 0·95 [95% CI 0·87–1·04], p=0·28), but did reduce cancer-related deaths after 5 years of follow-up in participants weighing less than 70 kg (appendix p 20).

**Figure 6:**
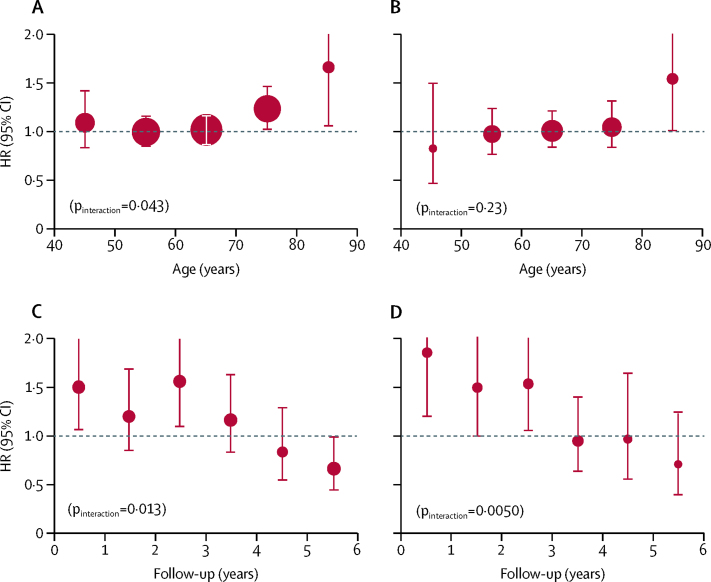
Effect of aspirin (all doses) versus control on early cancer risk in trials of aspirin in primary prevention of vascular events The size of the circles representing the point estimates of the HRs is proportional to the inverse of the variance of the estimate. (A) 3-year risk of cancer stratified by age. (B) 5-year risk of death due to cancer stratified by age. (C) Cancer risk stratified by year of follow-up in participants aged ≥70 years who had low weight for dose received (<70 kg for 75–100 mg and <80 kg for ≥325 mg). (D) Cancer risk stratified by year of follow-up in participants aged ≥70 years who had low height for dose received (1·6 m for 75–100 mg and <1·8 m for ≥325 mg). HR=hazard ratio.

Aspirin had no effect on the overall incidence of first cancer (3484 treated with aspirin *vs* 3448 treated with control; HR 1·01 [95% CI 0·96–1·06], p=0·64). However, aspirin appeared to increase cancer incidence in participants aged 70 years or older in the first 3 years of follow-up (1·20 [1·03–1·47], p=0·02), reflecting an apparent age-related hazard (p_interaction_=0·043; figure 6A). This increased risk in individuals aged 70 years or older was greatest in those of smaller body size (p_interaction_=0·034 for weight; p_interaction_=0·095 for height; appendix pp 21, 22), particularly in those weighing less than 70 kg (table 3) and, consequently, in women (143 women aged ≥70 years who were treated with aspirin had a first cancer within 3 years of follow-up *vs* 99 treated with control; HR 1·44 [95% CI 1·11–1·87], p=0·0069).

**Table 3:** Effect of aspirin versus control on risk of cancer during follow-up of primary prevention trials, stratified by dose, age, weight, period of follow-up, and diabetes status

		**Bodyweight <70 kg**				**Bodyweight ≥70 kg**			
		Aspirin	Control	HR (95% CI)	p value	Aspirin	Control	HR (95% CI)	p value
**Dose 75–100 mg**									
All follow-up									
	All participants	1352	1307	1·04 (0·96–1·12)	0·36	1430	1443	0·99 (0·92–1·07)	0·84
	Age <70 years	1039	1028	1·02 (0·93–1·11)	0·71	1251	1231	1·02 (0·94–1·10)	0·71
	Age ≥70 years	313	279	1·10 (0·93–1·30)	0·29	179	212	0·87 (0·71–1·07)	0·19
	Diabetes	147	111	1·31 (1·03–1·68)	0·031	138	134	1·04 (0·82–1·32)	0·75
<3 years' follow-up									
	All participants	530	439	1·21 (1·07–1·38)	0·0033	622	616	1·01 (0·90–1·13)	0·85
	Age <70 years	335	298	1·13 (0·97–1·33)	0·12	499	492	1·01 (0·89–1·15)	0·85
	Age ≥70 years	195	141	1·35 (1·09–1·69)	0·0071	123	124	1·01 (0·78–1·31)	0·91
	Diabetes	89	67	1·32 (0·96–1·81)	0·085	67	60	1·12 (0·79–1·59)	0·52
**All doses**									
All follow-up									
	All participants	1489	1421	1·04 (0·96–1·12)	0·36	1842	1804	1·00 (0·93–1·06)	0·89
	Age <70 years	1122	1103	1·01 (0·93–1·10)	0·80	1558	1517	1·00 (0·93–1·08)	0·96
	Age ≥70 years	367	318	1·11 (0·95–1·30)	0·18	284	287	0·96 (0·81–1·15)	0·68
	Diabetes	153	113	1·34 (1·05–1·71)	0·019	158	147	1·06 (0·85–1·33)	0·62
<3 years' follow-up									
	All participants	596	503	1·18 (1·04–1·33)	0·0089	859	808	1·03 (0·94–1·14)	0·51
	Age <70 years	373	338	1·10 (0·95–1·28)	0·21	671	640	1·01 (0·91–1·13)	0·84
	Age ≥70 years	223	165	1·31 (1·07–1·61)	0·009	188	168	1·09 (0·88–1·35)	0·45
	Diabetes	92	69	1·32 (0·97–1·81)	0·078	80	64	1·22 (0·88–1·70)	0·24

Analysis is limited to cancers diagnosed during the initial trial period. HR=hazard ratio.

The increased 3-year risk of cancer with aspirin in participants aged 70 years or older and weighing less than 70 kg was followed by a reduced incidence of cancer after 5 years in the analyses of all trials (appendix p 20). Figure 6 shows the time-course of risk of cancer in participants who were below the same dose–weight thresholds observed for the effect of aspirin on long-term risk of colorectal cancer (effective at <70 kg for 75–100 mg and <80 kg for ≥300 mg) and similar height thresholds (effective at <1·6 m for 75–100 mg and <1·8 m for ≥300 mg). Cancers increased by aspirin in participants below these thresholds were lower oesophageal or stomach cancer and colorectal cancer (weight below threshold: 83 *vs* 53; HR 1·48 [95% CI 1·05–2·09], p=0·027; height below threshold: 69 *vs* 33; 1·94, [1·27–2·96], p=0·0022).

The overall in-trial cancer incidence was increased by aspirin in people with diabetes who weighed less than 70 kg (table 3), with a similar hazard in those with diabetes who were 1·6 m or shorter, resulting in an increase in overall incidence in women with diabetes but not in men (appendix p 23). Risk of cancer was particularly high in women with diabetes who were younger than 50 years at baseline (28 of 1285 treated with aspirin *vs* six of 1283 treated with control; HR 4·35 [95% CI 1·80–10·5], p=0·0011), partly due to an increase in breast cancers, which was seen up to age 65 years (34 *vs* 13; 2·60 [1·13–2·57], p=0·012).

## Discussion

Suboptimal inhibition of thromboxane production has been shown with daily, low-dose aspirin in association with diabetes and obesity or high bodyweight.^5^, ^6^, ^7^, ^33^ Our findings that the clinical effectiveness of low-dose aspirin is reduced in people of greater weight and height, and that this association is reversed with higher doses, suggest the existence of a therapeutic window related to body size within which a given daily dose is most effective. Specifically, loss of efficacy can occur if the aspirin dose is too low or too high for body size, and other harms appear to result from excess dosing. Reductions in cardiovascular events and all-cause death at optimal doses for weight were substantial, highlighting the potential of more tailored aspirin dosing.

That low-dose aspirin at higher body size did not reduce cardiovascular events was consistent for daily and alternate-day dosing (figure 1), for all subgroups studied (appendix p 5), for early versus later follow-up (appendix pp 6, 7), for fatal cardiovascular events (appendix p 10), and for on-treatment analyses (appendix p 6). Loss of effect at larger body size, driven more by weight and height than by BMI, suggests insufficient systemic bioavailability of aspirin rather than increased platelet activation secondary to obesity. Acetylation of platelet COX-1 in the portal circulation alone might not fully account for the effect of aspirin on cardiovascular events, at least with daily dosing, and some systemic bioavailability might be required to inhibit COX-1 in megakariocytes,^7^ partially inhibit extra-platelet COX,^34^ or increase endothelial nitric oxide formation.^35^, ^36^ The bioavailability hypothesis is also supported by the somewhat greater loss of effect with enteric-coated or delayed-release aspirin than with daily standard-release aspirin in individuals weighing 70 kg or more (appendix p 9). However, the additional independent effect of smoking suggests that increased platelet activation also undermines low-dose aspirin, particularly with alternate-day dosing and in individuals with diabetes (table 2).

Weight-based dosing is commonly used for thrombolytics and intravenous antiplatelet treatment, but not for oral antiplatelet drugs. Our findings, therefore, have implications for practice. First, given that low-dose aspirin reduces cardiovascular events to a greater extent than previously thought in individuals weighing 50–69 kg, comparisons with other antiplatelet or antithrombotic regimens should be stratified by body size. Second, interactions between dose and weight probably explain previously reported sex differences in the effects of aspirin on risks of stroke and myocardial infarction,^9^, ^10^ which might not be explained by overweight or obesity alone. Third, that 75–100 mg aspirin was ineffective in primary prevention of cardiovascular events in the 80% of men and nearly 50% of women who weighed 70 kg or more in our study, even increasing the case fatality of first events, questions the use of once-daily low doses of aspirin irrespective of weight, particularly in people who smoke or are taking enteric-coated aspirin. More data on the weight dependence of the effects of standard-release, low-dose aspirin would be helpful, but in larger individuals, any advantage of enteric coating in reducing upper-gastrointestinal tract side-effects must be weighed against the loss of effect on cardiovascular events. Fourth, no trial of 75–100 mg aspirin versus placebo was available in the setting of secondary prevention of stroke or myocardial infarction (records were destroyed^37^ or weight or height were not recorded^32^). However because loss of effectiveness in primary prevention of stroke or myocardial infarction in participants weighing 70 kg or more was most evident at older ages, in people who smoked, and in people with diabetes (appendix p 5), similar findings are likely in long-term secondary prevention. Moreover, analyses of trials comparing antiplatelet regimens in secondary prevention of stroke show that dual antiplatelet treatment is more effective than low-dose aspirin alone in people weighing 70 kg or more (Rothwell, unpublished), further implying that platelet inhibition is suboptimal at higher weight. Inadequate inhibition might explain the paradoxically increased case fatality of cardiovascular events in people of higher weight who are on low-dose aspirin, particularly if COX-independent pathways are upregulated. Fifth, guidelines to target individuals at high predicted risk of vascular events (eg, people who are overweight and smoke) for primary prevention with low-dose aspirin might not yield benefits. Sixth, widespread use of 325 mg aspirin once a day in the USA, and elsewhere, is questionable in low-weight individuals given the effectiveness of lower doses and the apparent hazards of excess dosing. Seventh, the possible harm of 75–100 mg aspirin in individuals weighing less than 50 kg will be most relevant in Japan and in parts of Asia where bodyweight is often less than 50 kg in women. The effects of 25–30 mg aspirin twice a day in the ESPS-2 trial (figure 2) suggest that lower doses might be preferable for such women. The mechanism of harm from aspirin at very low bodyweight is uncertain, but systemic effects on underlying non-vascular disease might be relevant, particularly in those who are underweight. Finally, our findings might inform interpretation of trials of dual therapy with aspirin and P2Y12 inhibitors after coronary events. Previous trials^38^, ^39^ investigating this combination reported that the effects of irreversible inhibitors, such as clopidogrel and prasugrel, appeared to be more weight dependent than those of the reversible P2Y12 inhibitor (ticagrelor), possibly because of twice-daily dosing, and that the efficacy of clopidogrel versus ticagrelor when added to aspirin might depend on both aspirin dose and bodyweight.

We found that the effect of 75–100 mg aspirin on major bleeding was not lost until weight exceeded 90 kg. A 90 kg threshold was also found for reduced major bleeding on long-term treatment with 75 mg aspirin in a population-based cohort (OR for people weighing ≥90 kg was 0·58 [95% CI 0·36–0·84, p=0·002; Rothwell, unpublished).^40^ By contrast, we found that excess bleeding on high-dose aspirin did not diminish above 90 kg (appendix p 18), but appeared to increase with weight, similar to the effect on vascular events.

The effect of aspirin on long-term risk of colorectal cancer provided more evidence of interactions between dose and weight, with loss of effect for both vascular events and cancer with 75–100 mg aspirin in individuals weighing 70 kg or more. However, neither the loss of effect of higher-dose aspirin on risk of colorectal cancer in individuals weighing 80 kg or more nor the reduction in risk at lower weight was consistent with the effects on vascular events, and so the mechanisms of weight dependence might differ, possibly being more related to BMI than to lean bodyweight.

We confirmed that aspirin reduced the risk of in-trial death from cancer after 5 years' follow-up,^41^ but only in individuals weighing less than 70 kg, and stratification by body size identified other effects on short-term cancer risk. Possible increases in early risk of colorectal cancer and stomach cancers on high-dose aspirin have been noted previously,^13^, ^21^ but our findings in a much larger patient population suggest that low-dose aspirin might accelerate growth of some existing cancers at lower body size, particularly at older ages. The increased early risk should not necessarily be dismissed as simply an artifact of earlier diagnosis resulting from bleeding on aspirin. Although no increase in total cancers was observed during trial follow-up, there was no compensatory reduction in in-trial deaths from cancer at older ages that might result from earlier diagnosis, and neither the dependence of effects on body size nor the apparent increase in breast cancer in people with diabetes suggested a bleeding artifact. An adverse effect of salicylate, perhaps exacerbated at older ages because of reduced renal clearance of metabolites, could increase cancer growth.^42^ Furthermore, effects in people with diabetes might implicate insulin-related metabolic pathways and growth factors, or effects on extra-platelet COX.^34^ However, the findings first need to be confirmed or refuted in forthcoming trials. No increase in cancer risk was evident on 25 mg aspirin twice a day in the ESPS-2 trial (Rothwell, unpublished),^28^ and analyses of trials of other antiplatelet drugs are ongoing (Rothwell, unpublished).

The increased risk of sudden cardiac death on higher-dose aspirin was reported in some early trials,^43^ but was inconsistent,^44^ and there has been no systematic study or stratification by dose or body size. The suggestion of increased risk at lower bodyweight in people receiving 75–100 mg aspirin might be consistent with the increased risk of silent myocardial infarction in participants receiving 75 mg aspirin in the HOT trial,^24^ although our analysis was based on a small sample size and should be interpreted with caution. The fact that the hazard was mainly confined to use of higher-dose aspirin at lower body size might again be consistent with systemic COX-2 inhibition by aspirin, or possibly with other effects of salicylate.

Further research is required to validate and extend our findings. Three trials of low-dose aspirin in primary prevention, due to report in 2018, are studying groups in which we found effect modification by weight, including patients with diabetes (ASCEND; NCT00135226), people aged 70 years or older (ASPREE; NCT01038583), and people at increased vascular risk (ARRIVE; NCT00501059). Weight-stratified analyses of cardiovascular events are planned for the ARRIVE trial (NCT00501059) and also the JPPP trial^27^ (Rothwell, unpublished). Trials of twice-daily dosing are also required,^45^, ^46^ and some are ongoing (eg, ANDAMAN; NCT0252092). If lack of systemic bioavailability of daily aspirin undermines clinical effectiveness simply because the lack of inhibition of COX-1 in megakariocytes allows continued production of new uninhibited platelets, then effectiveness should be improved by more frequent dosing to inhibit these new platelets in the portal venous circulation, without the need for much higher doses. Our analysis of the ESPS-2 trial showed that 25 mg aspirin twice a day was highly effective in individuals weighing less than 70 kg (figure 2), and 50–100 mg twice a day might be effective in heavier individuals. Use of a low dose of aspirin twice a day might also reduce any hazards resulting from excess dosing. Trials (eg, ADAPTABLE; NCT02697916) of low versus higher doses of aspirin given once a day might be helpful if stratified by body size, but are otherwise still testing a one-dose-fits-all strategy. Future trials might study weight-dependent dosing. Previous trials of aspirin use in the short term to treat acute vascular events should be analysed to establish any weight dependence of acute effects, which might differ in the acute phase because of a reduced likelihood of missed doses. Trials of other antiplatelet drugs versus aspirin in longer-term prevention should also be assessed, although results might be complicated by weight-related dosing issues with some of these drugs.

Although our findings of dose–weight interactions in the effects of aspirin were consistent across trials and for vascular events and cancer, this study has some limitations. First, we included some older trials, and temporal changes in risk factors or medication could alter findings. However, low-dose, enteric-coated aspirin is widely used in clinical practice and was used in the three forthcoming primary prevention trials (ASPREE, ARRIVE, and ASCEND), so our main findings are likely to be relevant. Second, only one primary prevention trial^24^ used standard-release, low-dose aspirin, and no data were available from similar trials in secondary prevention;^32^, ^37^ thus, investigation of the weight dependence of the effect of standard-release, low-dose aspirin was inexact (appendix p 9). However, the aspirin component of the twice-daily tablet used in the ESPS-2 trial was standard release (figure 2). Third, some trials were only in men and some only in women, but we did analyses in both sexes separately when possible. Fourth, we validated findings in trials in secondary prevention of stroke and cannot be certain of the generalisability of the results to other secondary prevention settings. We also had no data on the different subtypes of ischaemic stroke. Atrial fibrillation is associated with greater height but was an exclusion criterion in the primary prevention trials, and onset of atrial fibrillation during follow-up would only account for a small proportion of strokes. Fifth, bodyweight might have changed during trial follow-up, although height is more stable than weight. Sixth, although we excluded confounding by withdrawal from randomised treatment (appendix p 6), we had no additional data on levels of compliance. It is possible that missed doses might particularly undermine effectiveness at larger body size. Seventh, optimal weight or height clearly differed for low-dose versus higher-dose aspirin, but differences between 300–325 mg and 500 mg or more could not be reliably ascertained, and no trials were available of doses between 100 mg and 300 mg. Eighth, body-fat distribution might modify the effect of aspirin to a greater extent than does BMI but is unlikely to confound the effect modification found for height and lean body mass. Finally, we did not aim to estimate the current overall balance of risk and benefit from aspirin given that several of the trials were done more than 20 years ago.

In conclusion, the optimal dose of aspirin to prevent cardiovascular events depends on bodyweight, driven more by lean body mass and height than by BMI. Low-dose (75–100 mg) aspirin once a day was ineffective in people weighing 70 kg or more, particularly in those who smoked or were treated with enteric-coated formulations, whereas higher doses became more effective with increasing weight. Given that the effects on sudden cardiac death and cancer also showed dose–weight interactions, the one-dose-fits-all strategy for daily aspirin use is unlikely to be optimal. The substantial reductions in cardiovascular events and death at optimal doses for weight highlight the potential to improve effectiveness and argue for a more tailored dosing strategy.
