# Supplementary material for: Effects of aspirin on risks of vascular events and cancer according to bodyweight and dose: analysis of individual patient data from randomised trials
Source: Lancet. 2018 Aug 4;392(10145):387–99. doi: 10.1016/S0140-6736(18)31133-4 (PMC6083400; doi:10.1016/S0140-6736(18)31133-4)
Supplement: Supplementary appendix [file mmc1.pdf]

THE LANCET

Supplementary appendix

This appendix formed part of the original submission and has been peer reviewed. We post it as supplied by the authors.

Supplement to: Rothwell PM, Cook NR, Gaziano JM, et al. Effects of aspirin on risks of vascular events and cancer according to bodyweight and dose: analysis of individual patient data from randomised trials. *Lancet* 2018; published online July 12. http://dx.doi.org/10.1016/S0140-6736(18)31133-4.

Webappendix

Effects of aspirin on risks of vascular events and cancer according to body weight and dose: Analysis of individual patient data from randomised trials

Peter M Rothwell, Nancy R. Cook, J Michael Gaziano, Jacqueline F Price, Jill F.F. Belch,
Maria Carla Roncaglioni, Takeshi Morimoto, Ziyah Mehta

P2. Characteristics of the seven trials of low-dose aspirin (75-100mg) vs control in primary prevention included in analyses of effect of aspirin on vascular events.

	**TPT[1]**	**HOT[2]**	**PPP[3]**	**JPAD[4]**	**POPADAD[5]**	**AAA[6]**	**WHS[7]**
Aspirin dose	75mg	75mg	100mg	81 or 100mg	100mg	100mg	100mg AD
Tablet formulation	Delayed-release	Standard	Enteric-coated	Enteric-coated	Enteric-coated	Enteric-coated	Standard
Patients (active / control)	2545 / 2540	9399/9391	2226/2269	1262/1277	638/638	1675/1675	19,934/19,942
Placebo-control /double-blind	Yes	Yes	No	No	Yes	Yes	Yes
Recruitment period	1989 - 1992	1992 - 1994	1994-1998	2002-2004	1997-2001	1998-2001	1992-1995
Year completed	1997	1997	1998	2008	2006	2008	2004
Mean (SD) age at randomisation	57.5 (6.7)	61.5 (7.5)	64.4 (7.6)	64.5 (10.0)	60.3 (10.0)	62.0 (6.6)	54.6 (7.0)
% Male	100%	53%	42.5%	54.6%	44.1%	28.5%	0%
Current smokers	41.2%	15.9%	14.8%	21.2%	31.7%	32.4%	13.1%
Mean (SD) body weight (kg)	82.5 (12.4)	79.7 (14.9)	73.5 (13.5)	61.1 (11.3)	82.3 (17.1)	na	70.2 (14.5)
Median (range) weight (kg)	81.2 (40.5-156.0)	78.6 (32.0-190.5)	72.0 (38.0-160.0)	60.0 (32.5 -123.0)	80.0 (45.0-149.0)	na	68.0 (38.1-193.2)
Data on weight missing: n (%)	0 (0%)	6 (0.03%)	10 (0.22%)	4 (0.16%)	1 (0.08%)	100%	16 (0.04%)
Mean (range) duration of scheduled treatment (years)	6.9 (4.3-8.6)[1]	3.8 (3.3-4.9)	3.6 (1.0-5.2)	4.4 (3.0-5.4)[1]	6.7 (4.5-8.6)[1]	8.2 (6.7-10.5)	10.1 (8.2-10.9)
Method and frequency of clinical follow-up	Face-to-face annually, record review every 6m	Face-to-face at 3m, 6m and then every 6 months	Face-to-face annually	Face-to-face every 2-4 weeks	Face-to-face every 6 months	Face-to-face at 3m, 1 year and 5 years; telephone annually	Annual questionnaire; outcomes confirmed by medical record review

1. Medical Research Council's General Practice Research Framework. Thrombosis prevention trial: randomised trial of low-intensity oral anticoagulation with warfarin and low-dose aspirin in the primary prevention of ischaemic heart disease in men at increased risk. Lancet 1998; 351:233-41.

2. Hansson L, Zanchetti A, Carruthers SG, Dahlof B, Elmfeldt D, Julius S, Menard J, Rahn KH, Wedel H, Westerling S. Effects of intensive blood-pressure lowering and low-dose aspirin in patients with hypertension: principal results of the Hypertension Optimal Treatment (HOT) randomised trial. HOT Study Group. Lancet 1998; 351:1755-62.

3. Collaborative Group of the Primary Prevention Project. Low-dose aspirin and vitamin E in people at cardiovascular risk: a randomised trial in general practice. Lancet 2001; 357: 89-95.

4. Ogawa H, Nakayama M, Morimoto T, Uemura S, Kanauchi M, et al. Japanese Primary Prevention of Atherosclerosis With Aspirin for Diabetes (JPAD) Trial Investigators. Low-dose aspirin for primary prevention of atherosclerotic events in patients with type 2 diabetes: a randomized controlled trial. JAMA 2008; 300: 2134-41.

5. Belch J, MacCuish A, Campbell I, Cobbe S, Taylor R, Prescott R, Lee R, et al; Prevention of Progression of Arterial Disease and Diabetes Study Group; Diabetes Registry Group; Royal College of Physicians Edinburgh. The prevention of progression of arterial disease and diabetes (POPADAD) trial: factorial randomised placebo controlled trial of aspirin and antioxidants in patients with diabetes and asymptomatic peripheral arterial disease. BMJ 2008; 337:a1840. doi: 10.1136/bmj.a1840.

6. Fowkes FG, Price JF, Stewart MC, Butcher I, Leng GC, et al. Aspirin for Asymptomatic Atherosclerosis Trialists. Aspirin for prevention of cardiovascular events in a general population screened for a low ankle brachial index: a randomized controlled trial. JAMA 2010; 303: 841-8.

7. Ridker PM, Cook NR, Lee IM, Gordon D, Gaziano JM, Manson JE, Hennekens CH, Buring JE. A randomized trial of low-dose aspirin in the primary prevention of cardiovascular disease in women. N Engl J Med 2005; 352: 1293-304.

P3. Characteristics of trials of other doses of aspirin in primary prevention of vascular events and in secondary prevention of stroke.
All trials are of aspirin versus control apart from the Dutch TIA Aspirin trial which compared two different doses of aspirin.
None of the trials used enteric-coated or delayed-release aspirin routinely, although enteric-coated aspirin could be requested in BDAT.

	**Primary prevention of vascular events**		**Secondary prevention after TIA or ischaemic stroke**			
	**British Doctors Aspirin Trial (BDAT)[1]**	**Physicians Health Study (PHS)[2]**	**UK-TIA Aspirin Trial[3]**	**European Atrial Fibrillation Trial[4]**	**Dutch-TIA Aspirin Trial[5]**	**European Stroke Prevention Trial 2[6]**
Aspirin dose	500mg	325mg AD	300mg /1200mg	300mg	283mg vs 30mg	25mg BD
Participants (active/control)	3429 / 1710	11,037 / 11,034	811 / 821 / 817	404/378	1576/1555	3299/3303
Placebo control/ double-blind	No	Yes	Yes	Yes	Yes	Yes
Recruitment period	1978 & 1979	1981-1984	1979 - 1985	1989-1992	1986-1989	1988-1993
Year completed	1984	1988	1986	1993	1990	1995
Mean (SD) age at randomisation	61.6 (7.0)	53.8 (9.6)	60.3 (9.0)	73.0 (8.0)	65.1 (10.0)	66.7 (11.1)
% Male	100%	100%	73.0%	56.2	65.3%	58.0%
Current smokers	31.0%	11.0%	53.0%	19.1%	44.5%	24.1%
Mean (SD) body weight	75.6 (12.1)	79.0 (10.6)	72.4 (12.1)	70.9 (13.1)	74.6 (11.7)	71.9 (12.7)
Median (range) body weight	76.2 (44 -127)	78.0 (47 -170)	72.0 (38 -129)	70.0 (36 – 150)	74.0 (38-197)	71.0 (35 – 133)
Data on weight missing: n (%)	54 (1.05%)	4 (0.02%)	13 (0.53%)	6 (0.77%)	0 (0%)	0 (0%)
Median (range) duration of scheduled treatment (years)	6.0 (5.0 – 6.0)	5.1 (3.8-6.4)	4.4 (1.0 – 7.1)	2.6 (1.0-4.5)	2.6 (1.0-4.3)	2.0 (2.0-2.0)
Method of in-trial follow-up	Questionnaire every 6 months	Annual questionnaire	Face-to-face every 4 months	Face-to-face every 4 months	Face-to-face every 4 months	Face-to-face every 3 months

1. Peto R, Gray R, Collins R, Wheatley K, Hennekens C, Jamrozik K, Warlow C, Hafner B, Thompson E, Norton S. Randomised trial of prophylactic daily aspirin in British male doctors. BMJ 1988; 296: 313-316
2. Steering Committee of the Physicians' Health Study Research Group. Final report on the aspirin component of the ongoing Physicians' Health Study. N Engl J Med 1989; 321: 129-35.
3. Farrell B, Godwin J, Richards S, Warlow C. The United Kingdom transient ischaemic attack (UK-TIA) aspirin trial: final results. J Neurol Neurosurg Psychiatry 1991;54:1044-1054
4. European Atrial Fibrillation Trial Study Group. Secondary prevention in non-rheumatic atrial fibrillation after transient ischaemic attack or minor stroke. Lancet 1993;342:1255-62.
5. Dutch TIA Trial Study Group. A comparison of two doses of aspirin (30mg vs 283mg a day) in patients after a transient ischemic attack or minor ischemic stroke. N Engl J Med 1991;325:1261-6
6. ESPS 2 Group. European stroke prevention study: 2. Efficacy and safety data. J Neurol Sci 1997; 151(suppl): S1-77.

NOTE: The EDTRS trial (JAMA 1992; 268: 1292-1300) was not eligible as a primary prevention trial as a significant proportion of participants had had vascular events prior to randomisation.

P4. Effect of low-dose aspirin versus control in primary prevention on risk of all cardiovascular events according to body weight in participants not randomised to also receive vitamin E compared with those who were randomised to receive vitamin E in the three factorial design trials of aspirin and vitamin E.[1-3] Error bars are 95%CI and size of the point estimate is proportional to the inverse of the variance.

Note: The apparently reduced benefit of aspirin at 50-69kg in the group allocated vitamin E appeared to be due to a reduction in risk of ischaemic events by vitamin E in the placebo groups of the aspirin comparison without any further reduction by combination treatment in the aspirin-treated group.

No vitamin E **Allocated vitamin E**

Alternate-day 100mg

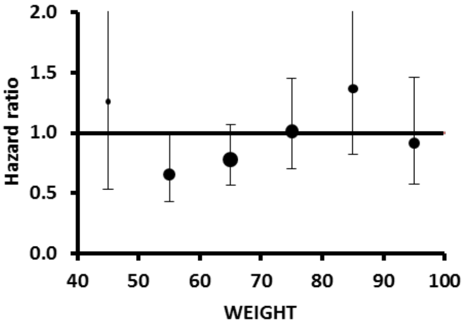

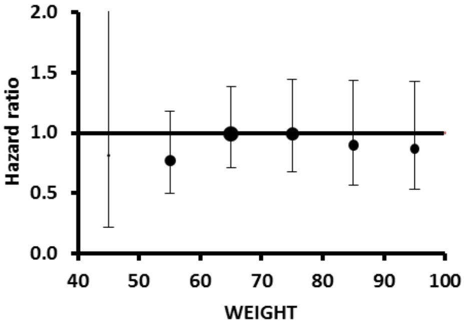

All 75-100mg

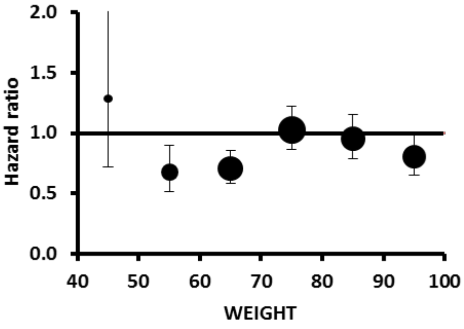

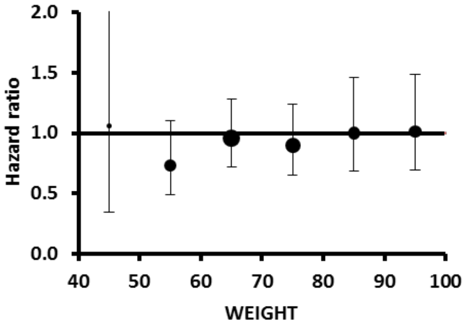

1. Ridker PM, Cook NR, Lee IM, Gordon D, Gaziano JM, Manson JE, Hennekens CH, Buring JE. A randomized trial of low-dose aspirin in the primary prevention of cardiovascular disease in women. N Engl J Med 2005; 352: 1293-304.

2. Belch J, MacCuish A, Campbell I, Cobbe S, Taylor R, Prescott R, Lee R, et al; Prevention of Progression of Arterial Disease and Diabetes Study Group; Diabetes Registry Group; Royal College of Physicians Edinburgh. The prevention of progression of arterial disease and diabetes (POPADAD) trial: factorial randomised placebo controlled trial of aspirin and antioxidants in patients with diabetes and asymptomatic peripheral arterial disease. BMJ 2008; 337:a1840. doi: 10.1136/bmj.a1840.

3. Collaborative Group of the Primary Prevention Project. Low-dose aspirin and vitamin E in people at cardiovascular risk: a randomised trial in general practice. Collaborative Group of the Primary Prevention Project. Lancet 2001; 357: 89-95.

P5. Effect of low-dose aspirin vs control in primary prevention on risk of CV-events stratified by weight and other baseline clinical characteristics. For comparability the analysis by sex is confined to aspirin only vs placebo only (i.e. excludes those randomised to vitamin E). Error bars are 95%CI and size of the point estimate is proportional to the inverse of the variance.

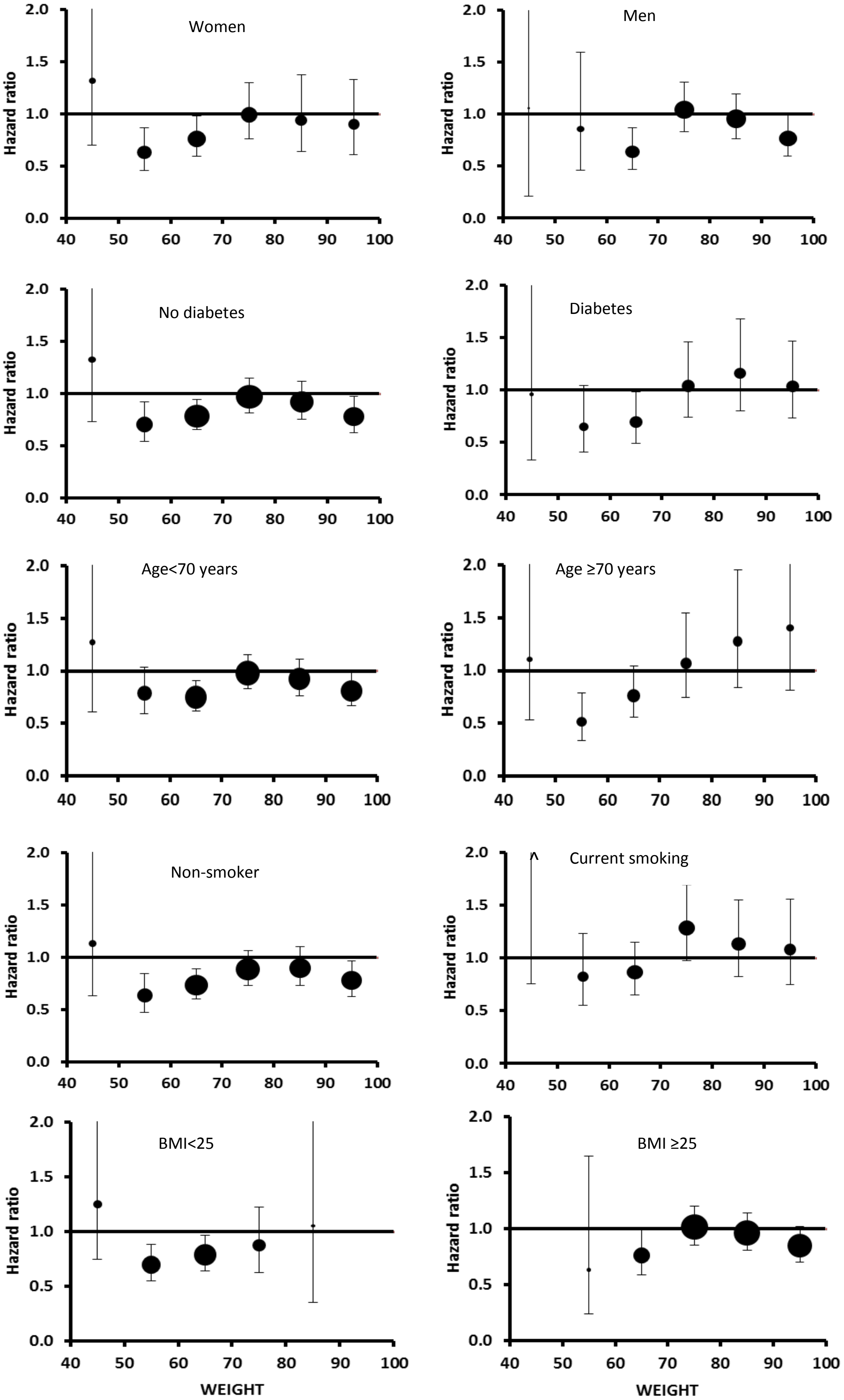

P6. Pooled analyses of the effect of aspirin 75-100mg vs control in primary prevention on risks of ischaemic stroke and of all CV events at weight 50-69kg vs higher weight by intention to treat and also with censoring at time of discontinuation of trial treatment. The thicker line represents the aspirin groups and the thinner line the placebo groups.

Ischaemic stroke | **All CV events**

Weight 50-69kg

Intention to treat analysis

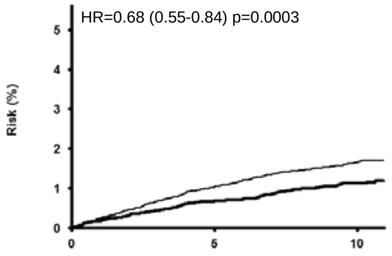

Placebo	15270	11057	6860
Aspirin	15242	11140	6891

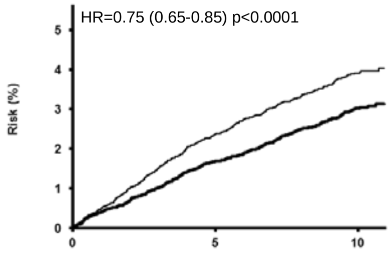

15270	11006	6811
15242	11093	6839

Censored at treatment discontinuation

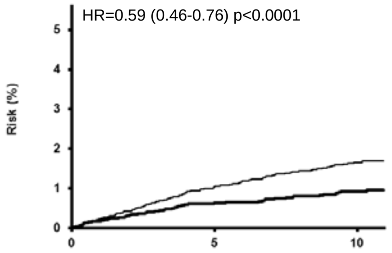

Placebo	15270	7563	3790
Aspirin	15242	7456	3662

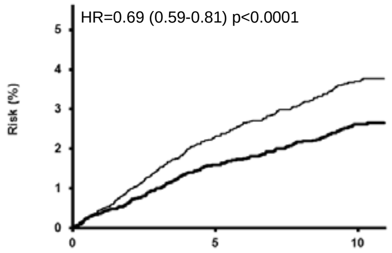

15270	7552	3779
15242	7446	3651

Weight ≥70kg

Intention to treat analysis

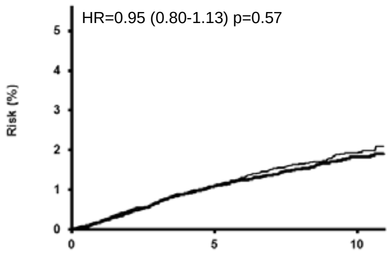

Placebo	19792	10586	5324
Aspirin	19750	10546	5374

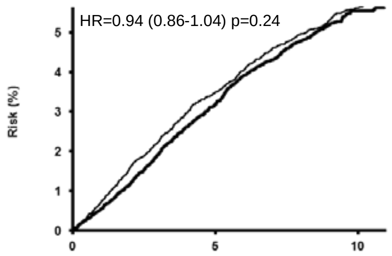

19792	10444	5263
19750	10440	5308

Censored at treatment discontinuation

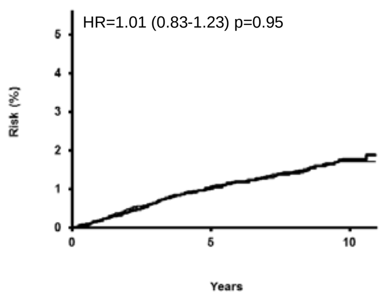

Placebo	19792	6762	2692
Aspirin	19750	6570	2602

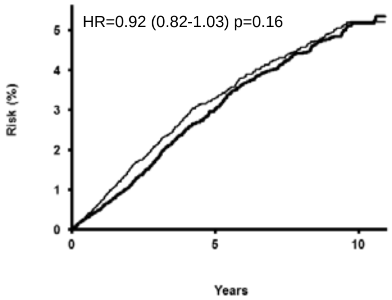

19792	6740	2680
19750	6548	2583

P7. Effect of aspirin 75-100mg versus control in primary prevention on risk of cardiovascular events and major bleeds according to body weight and period of follow-up. Error bars are 95%CI and size of the point estimate is proportional to the inverse of the variance.

All cardiovascular events **Major bleeds**

Follow-up period

0-3 years

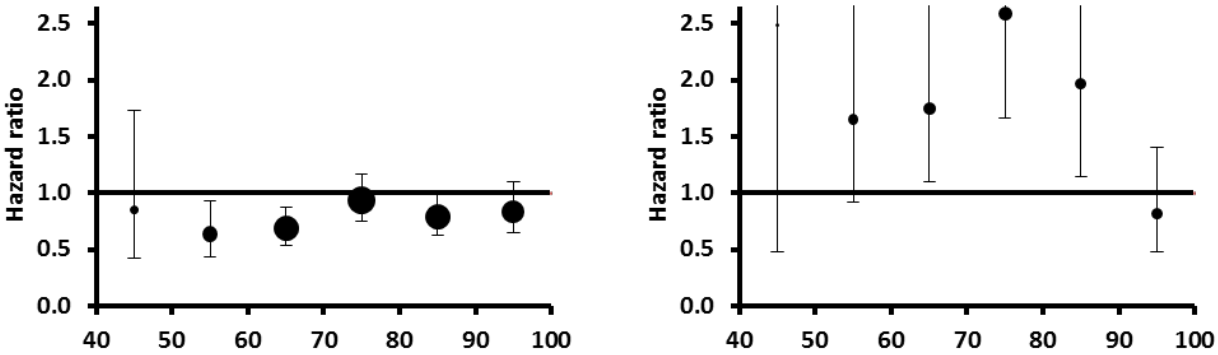

≥3 years

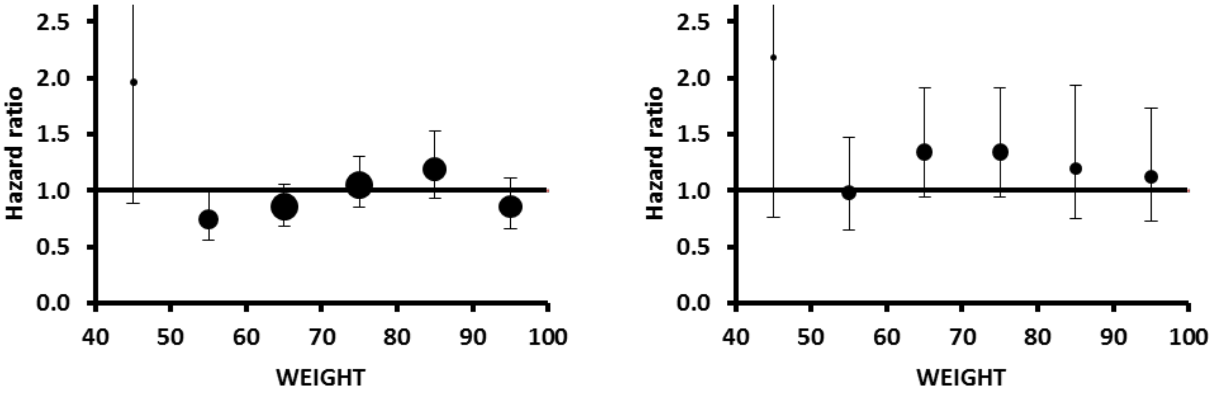

P8. Effect of aspirin 75-100mg versus control in primary prevention on risk of cardiovascular events in each trial according to weight≥70kg and current smoking (0=neither; 1= either; 2=both). The HOT trial was the only trial that used a standard-release non-enteric-coated aspirin formulation.

		Treatment A vs P	HR (95% CI)	Interaction
<u>Trials</u>:				
**WHS**				
	0	142 vs 197	0.72 (0.58-0.89)	
	1	257 vs 267	0.96 (0.81-1.14)	0.0007
	2	72 vs 53	1.40 (0.98-2.00)	
**HOT**				
	0	60 vs 76	0.81 (0.58-1.13)	
	1	212 vs 247	0.84 (0.70-1.01)	0.62
	2	50 vs 55	0.92 (0.63-1.35)	
**TPT**				
	0	15 vs 18	0.72 (0.36-1.44)	
	1	103 vs 143	0.71 (0.55-0.92)	0.036
	2	90 vs 84	1.11 (0.82-1.49)	
**PPP**				
	0	13 vs 29	0.45 (0.23-0.86)	
	1	26 vs 27	0.97 (0.57-1.67)	0.13
	2	7 vs 10	0.80 (0.30-2.09)	
**JPAD**				
	0	26 vs 35	0.78 (0.47-1.29)	
	1	18 vs 19	0.92 (0.48-1.75)	0.27
	2	3 vs 0	---	
**POPADAD**				
	0	19 vs 19	0.86 (0.45-1.62)	
	1	58 vs 75	0.81 (0.58-1.15)	0.047
	2	38 vs 20	1.75 (1.02-3.01)	

Effect of aspirin 75-100mg versus control in primary prevention on risk of cardiovascular events in each trial according to weight 50-69kg versus ≥70kg. The HOT trial was the only trial that used a standard-release non-enteric-coated aspirin formulation.

WEIGHT 50-69kg

HOT	0.77	0.57-1.04
JPAD	0.76	0.47-1.24
POPADAD	0.74	0.44-1.23
PPP	0.49	0.27-0.92
TPT	0.50	0.31-0.81
WHS	0.81	0.67-0.97
**TOTAL**	0.75	0.65-0.85

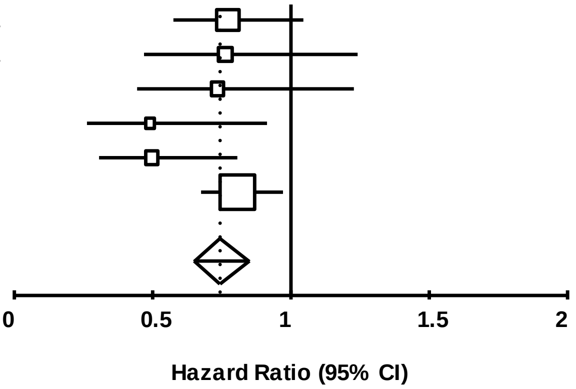

Significance: p = <0.0001

Heterogeneity: p = 0.39

WEIGHT ≥ 70kg

HOT	0.85	0.72-1.02
JPAD	1.55	0.59-4.07
POPADAD	1.08	0.80-1.47
PPP	0.92	0.56-1.50
TPT	0.92	0.76-1.13
WHS	0.99	0.83-1.19
**TOTAL**	0.94	0.86-1.04

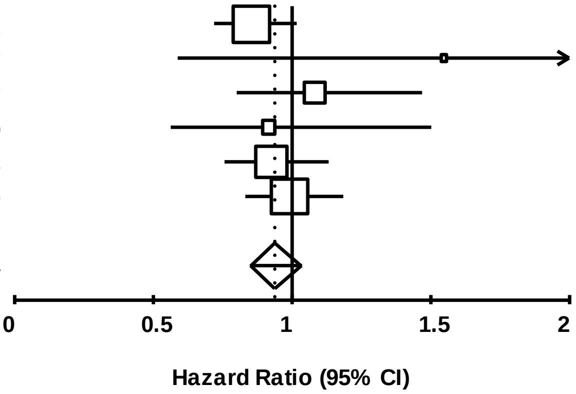

Significance: p = 0.23

Heterogeneity: p = 0.63

P9. Effect of aspirin 75-100mg versus control in primary prevention on risk of all cardiovascular events stratified by the aspirin tablet formulation and dosing frequency. The delayed-release formulation used in the Thrombosis Prevention Trial is included with enteric-coated preparations in the analysis. Error bars are 95%CI and size of the point estimate is proportional to the inverse of the variance.

Enteric-coated/delayed-release (daily)

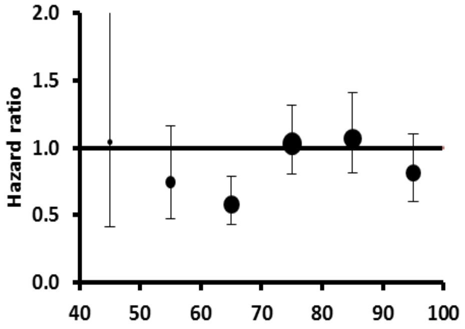

Standard release (daily or alternate-day)

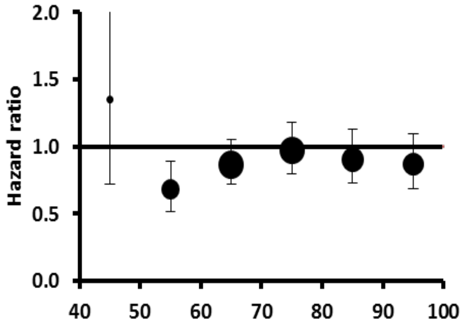

Standard release 100mg alternate-day

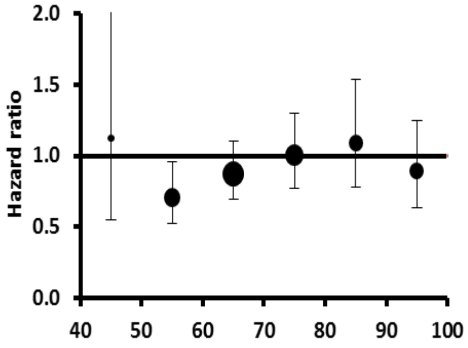

Standard release 75mg daily

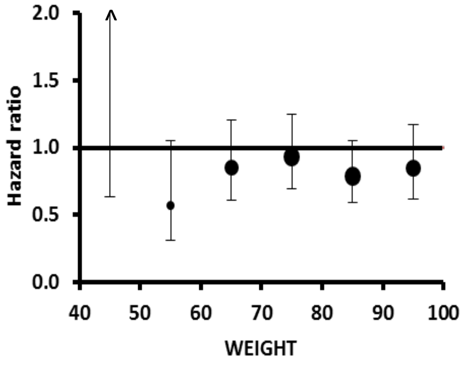

P10. Effect of aspirin 75-100mg vs control in primary prevention on risk of all first vascular events, fatal events, and case-fatality of first events stratified according to weight. Error bars are 95%CI and size of the point estimate is proportional to the inverse of the variance.

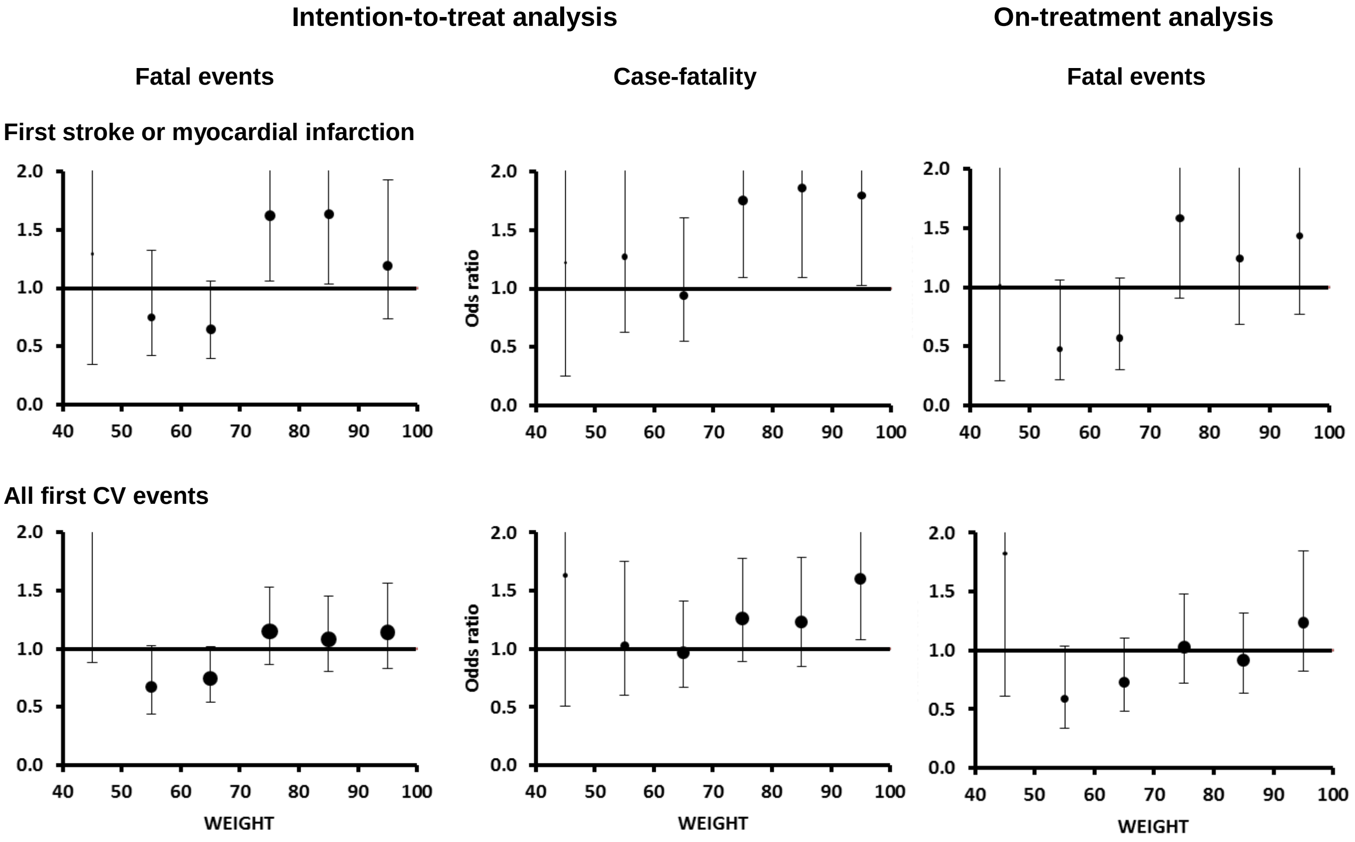

Effect of 75-100mg aspirin versus control in primary prevention of all cardiovascular events and all cardiovascular death according to weight comparing intention-to-treat analysis with on-treatment analysis.

	Intention to treat		On treatment	
**Weight**	**HR (95% CI)**	**p**	**HR (95% CI)**	**p**
**ALL CV EVENTS**				
<70kg	0.77 (0.68-0.87)	<0.0001	0.72 (0.62-0.83)	<0.0001
≥70kg	0.94 (0.86-1.04)	0.24	0.92 (0.82-1.03)	0.16
**CV DEATH**				
<70kg	0.79 (0.63-1.00)	0.048	0.73 (0.54-1.01)	0.054
≥70kg	1.09 (0.93-1.29)	0.30	1.02 (0.83-1.27)	0.83

P11. Effect of aspirin versus control in primary prevention on risk of major bleeding according to weight (top). The lower graphs also include the ESPS-2 trial. Analyses include intracerebral haemorrhage and major extracranial bleeding. The ’no factorial’ analysis includes only patients randomised to aspirin only versus placebo only (i.e. excluding the arms of those factorial design trials in which patients also received vitamin E, warfarin, or dipyridamole). Error bars are 95%CI and size of the point estimate is proportional to the inverse of the variance.

Primary prevention trials only

All low-dose

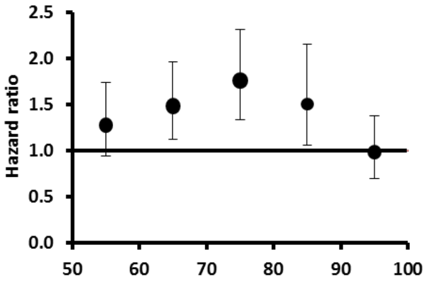

Alternate-day low dose

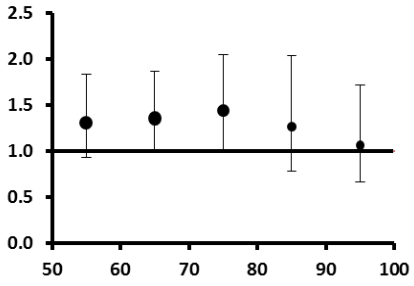

Daily low-dose

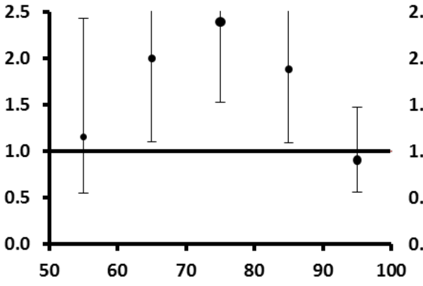

Low dose (no factorial)

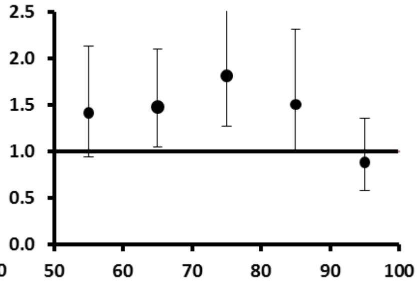

All trials

All low-dose

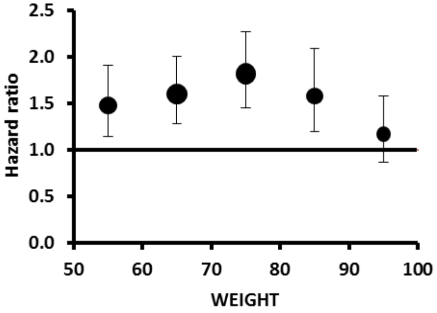

Low-dose: age <70 years

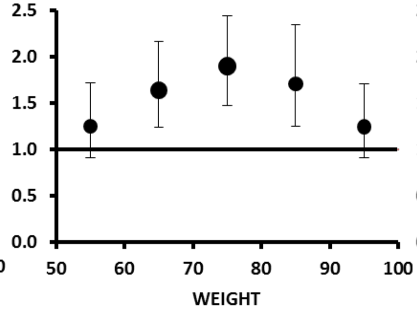

Low-dose: age ≥70 years

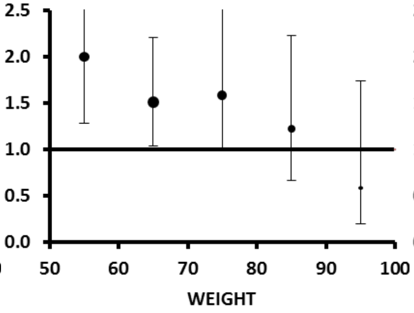

Low-dose (no factorial)

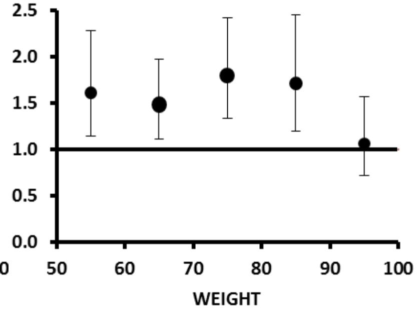

P12.

Effect of aspirin versus control in primary prevention of cardiovascular events in the tallest participants (top quintile of height) versus shorter participants (lower four quintiles) stratified by dose.

In all of the low-dose aspirin trials, height was recorded in metres and the following sex-specific thresholds were used for analysis: women - shorter <1.70m vs tall ≥1.70 (top quintile); men - shorter ≤1.80m vs tall >1.80m (top quintile). Both high-dose aspirin trials were done in men only and height was recorded in feet and inches and the following thresholds were closest to the quintile boundary: shorter <6 feet; tall ≥6 feet.

For presentation in the table the number of outcomes in the BDAT placebo group are doubled in view of the 2:1 randomisation ratio, but the actual numbers were used in all statistical analyses.

**Height:**		**Shorter**			**Tallest**		
**Outcome**	**Asa v pla**	**HR (95% CI)**	**p**	**Asa v pla**	**HR (95% CI)**	**p**	**p (interaction)**
**75-100mg trials**							
**Stroke**	419 v 519	0.81 (0.71-0.92)	0.0012	81 v 62	1.30 (0.93-1.81)	0.12	0.0085
**Myocardial infarct**	455 v 549	0.83 (0.73-0.94)	0.0027	89 v 75	1.19 (0.87-1.61)	0.27	0.032
**Vascular death**	346 v 374	0.93 (0.80-1.07)	0.32	78 v 56	1.38 (0.98-1.95)	0.063	0.036
**All CV events**	1002 v 1205	0.83 (0.76-0.90)	<0.0001	206 v 168	1.22 (1.00-1.50)	0.055	0.0006
Female	540 v 651	0.83 (0.74-0.93)	0.001	127 v 107	1.23 (0.95-1.59)	0.12	
Male	462 v 554	0.83 (0.74-0.94)	0.0037	79 v 61	1.19 (0.85-1.66)	0.31	
All CV events (BMI<30)	761 v 900	0.84 (0.76-0.92)	0.0004	153 v 132	1.15 (0.91-1.45)	0.23	0.014
**≥325mg trials**							
**Stroke**	174 v 135	1.26 (0.99-1.61)	0.065	36 v 47	0.87 (0.55-1.38)	0.55	0.16
**Myocardial infarct**	231 v 309	0.71 (0.59-0.85)	0.0002	40 v 73	0.55 (0.37-0.82)	0.0033	0.26
**Vascular death**	201 v 173	1.15 (0.91-1.44)	0.25	35 v 70	0.55 (0.36-0.85)	0.0068	0.0032
**All CV events**	505 v 494	0.98 (0.85-1.12)	0.72	99 v 162	0.66 (0.51-0.85)	0.0017	0.0083
PHS (325mg)	251 v 276	0.90 (0.76-1.07)	0.23	65 v 96	0.68 (0.50-0.93)	0.017	
BDAT (500mg)	254 v 218	1.14 (0.91-1.42)	0.26	34 v 66	0.56 (0.35-0.90)	0.017	
All CV events (BMI<30)	469 v 457	0.99 (0.86-1.13)	0.85	94 v 151	0.67 (0.51-0.88)	0.0034	0.012

Effect of aspirin versus control in on risk of sudden cardiac death with doses above data-derived weight thresholds (75-100mg at <50kg; 325mg at <70kg; 500mg at <90kg) – as reported in the Results section of the main paper.

	**Events/patients: aspirin vs placebo**	**HR (95% CI)**	**p**	**p(int)**
Excess dose	82/8488(0.97%) vs 27/6838(0.39%)	2.03 (1.31-3.15)	0.0015	0.0018
Not excess dose	246/41853(0.59%) vs 256/41878(0.61%)	0.96 (0.80-1.14)	0.62	

P13. Effect of aspirin 75-100mg versus control in primary prevention on for risk of cardiovascular events stratified by lean body mass, fat mass and body surface area and frequency of aspirin (alternate-day; daily or alternate-day).
Error bars are 95%CI and size of the point estimate is proportional to the inverse of the variance.

100mg alternate-day | **All 75-100mg**

Lean body mass (kg)

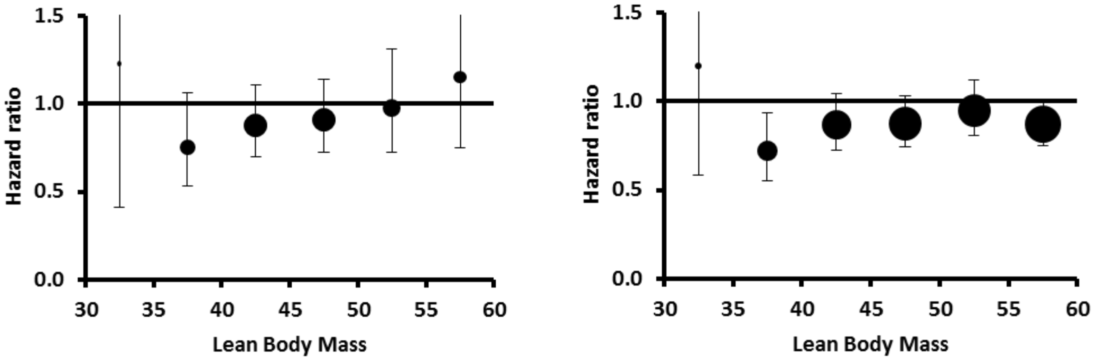

Fat mass (kg)

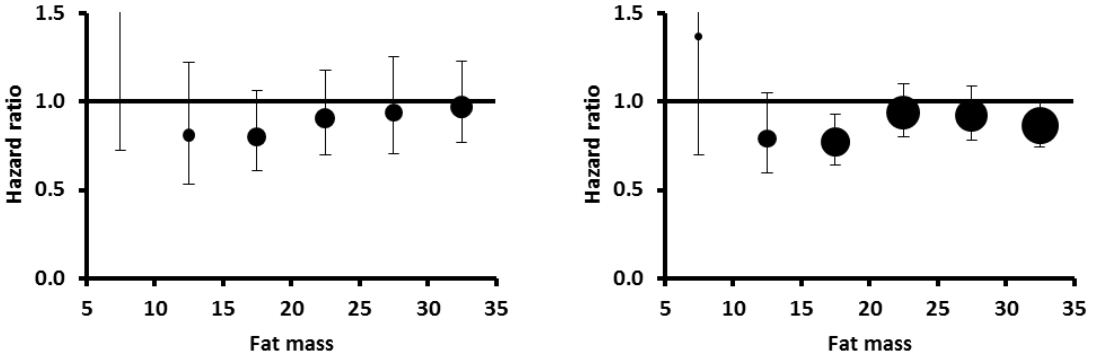

Body surface area (m^2)

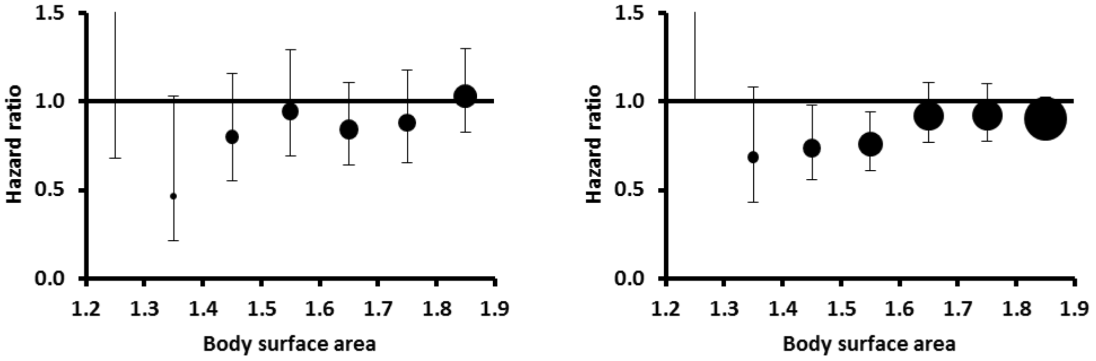

Estimated **lean body mass** (based on weight in kg and height in cm) was based on the Hume formula:[1]
Male = (0.32810 x weight) + (0.33929 x height) - 29.5336;
Female = (0.29569 x weight) + (0.41813 x height) - 43.2933.

Estimated **fat mass:** total body weight minus lean body mass.

Estimated **body surface area** was based on the Du Bois formula:[2] BSA = 0.007184 x weight$^{0.425}$ x height $^{0.725}$

1. Hume, R. Prediction of lean body mass from height and weight. Journal of Clinical Pathology 1966; 19: 389–91

2. Du Bois D, Du Bois EF. A formula to estimate the approximate surface area if height and weight be known. Arch Int Med 1916; 17: 863–71.

P14. Effect of aspirin 75-100mg versus control in primary prevention on risk of stroke and on risk of all cardiovascular events according to body size parameters.

	Events: aspirin vs placebo	HR (95% CI)	P	
**All stroke**				*Interaction*
BMI				
<25	169 vs 229	0.73 (0.60-0.89)	0.0021	0.078
≥25-29	214 vs 213	1.01 (0.83-1.22)	0.94	
≥30	117 vs 139	0.85 (0.66-1.08)	0.19	
Body weight				
<70	191 vs 269	0.71 (0.59-0.85)	0.0002	0.0058
≥70	309 vs 312	1.00 (0.85-1.16)	0.95	
Lean body mass				
<50	274 vs 363	0.75 (0.64-0.88)	0.0004	0.01
≥50	226 vs 218	1.04 (0.86-1.25)	0.69	
Fat mass				
<25	262 vs 319	0.82 (0.70-0.97)	0.018	0.41
≥25	238 vs 262	0.91 (0.76-1.08)	0.28	
Body surface area				
<1.8	231 vs 311	0.74 (0.62-0.88)	0.0005	0.014
≥1.8	269 vs 270	1.00 (0.84-1.18)	0.99	
**All cardiovascular events**				
BMI				
<25	384 vs 473	0.80 (0.70-0.91)	0.0010	0.19
≥25-29	530 vs 559	0.95 (0.84-1.07)	0.36	
≥30	294 vs 341	0.87 (0.74-1.02)	0.077	
Body weight				
<70	404 vs 524	0.76 (0.67-0.86)	<0.0001	0.0072
≥70	806 vs 853	0.95 (0.86-1.04)	0.28	
Lean body mass				
<50	583 vs 695	0.83 (0.75-0.93)	0.0013	0.22
≥50	625 vs 678	0.92 (0.83-1.03)	0.14	
Fat mass				
<25	602 vs 692	0.86 (0.77-0.96)	0.0074	0.67
≥25	606 vs 681	0.89 (0.80-0.99)	0.039	
Body surface area				
<1.8	485 vs 597	0.81 (0.71-0.91)	0.0004	0.067
≥1.8	723 vs 776	0.93 (0.84-1.03)	0.18	

P15.

Effect of aspirin (25mg twice daily) versus placebo on risk of all cardiovascular (CV) events and on fatal CV-events stratified by weight and by period of follow-up in the ESPS-2 trial. Error bars are 95%CI and size of the point estimate is proportional to the inverse of the variance.

All CV events **Fatal CV event**

All follow-up

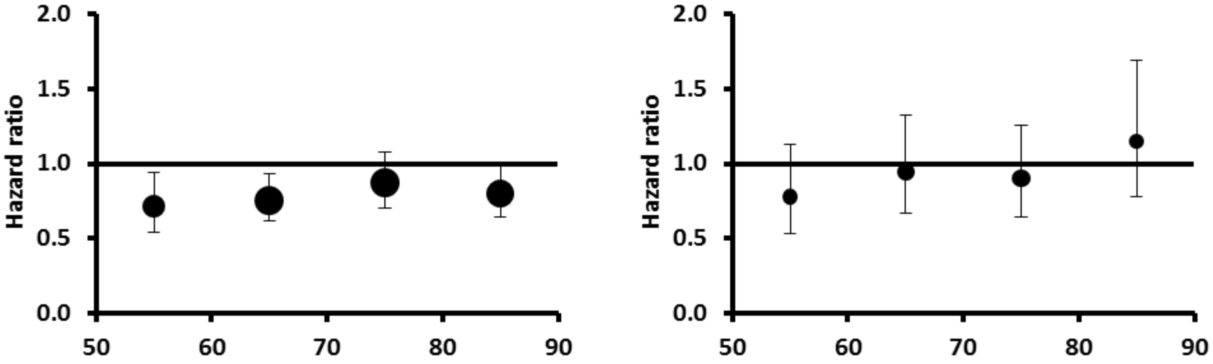

Acute phase (<90 days)

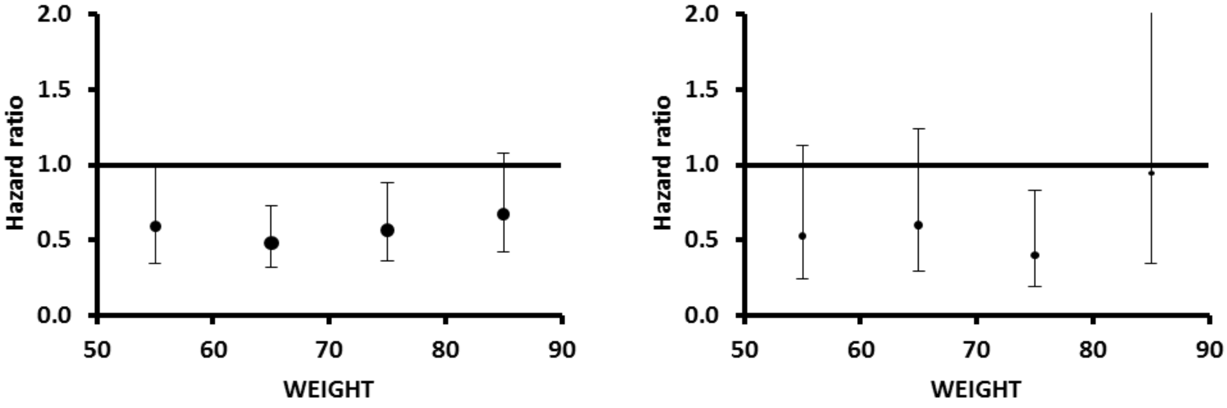

Effect of aspirin (25mg twice daily) versus placebo on risk of all cardiovascular (CV) events and on all CV events or death in women during all follow-up in the ESPS-2 trial stratified by weight: additional detail on results given in the Results section of the main paper.

CV events

Weight	Events/patients: aspirin vs placebo	HR (95% CI)	p	p(int)
<70	141/843(16.73%) vs 202/876(23.06%)	0.67 (0.54-0.84)	0.0003	0.033
≥70	87/544(15.99%) vs 81/511(15.85%)	1.01 (0.75-1.37)	0.95	

CV events or death

Weight	Events/patients: aspirin vs placebo	HR (95% CI)	p	p(int)
<70	167/843(19.81%) vs 243/876(27.74%)	0.66 (0.55-0.81)	<0.0001	0.014
≥70	100/544(18.38%) vs 92/511(18.00%)	1.02 (0.77-1.36)	0.89	

Figure 2 of main paper shows a significant reduction in risk of all CV events and death on aspirin versus placebo in ESPS-2 in women weighing <50kg based on the following numbers of events/participants: 23/95 (aspirin group) versus 35/81 (placebo group).

P16. Effect of high-dose aspirin versus control on risk of all cardiovascular events stratified by weight, trial setting, aspirin dose (stratified by trial). P-trend with weight as continuous variable.
N is given for the total number of events (note the 2:1 randomisation in BDAT and UKTIA). However, in UKTIA, the placebo group is represented twice in the analyses by dose (given the two different dose groups in UKTIA), in dose 300-325mg and then again in dose ≥500mg.

	**Trial-specific analysis**				**Pooled analysis**		
**Weight (kg)**	**N**	**HR (95% CI)**	**p**	**Weight (kg)**	**N**	**HR (95 % CI)**	**p**
**Primary prevention**				**Primary prevention**			
**PHS**				<60	27	1.72 (0.74-3.98)	0.21
<70	132	0.91 (0.65-1.29)	0.6	60-69	224	0.94 (0.72-1.23)	0.65
70-79	258	0.89 (0.70-1.14)	0.36	70-79	444	0.99 (0.82-1.19)	0.89
≥80	299	0.78 (0.62-0.98)	0.034	80-89	270	0.80 (0.62-1.01)	0.063
**BDAT**				≥90	154	0.73 (0.53-1.00)	0.051
<70	119	1.15 (0.78-1.69)	0.48	*Trend*		*P=0.017*	
70-79	186	1.14 (0.83-1.57)	0.4	**All trials**			
≥80	125	0.75 (0.53-1.08)	0.12	<60	157	1.21 (0.90-1.62)	0.22
**Subtotal**				60-69	424	0.89 (0.74-1.06)	0.19
<70	251	1.01 (0.79-1.30)	0.92	70-79	725	0.87 (0.76-1.01)	0.062
70-79	444	0.98 (0.81-1.19)	0.84	80-89	399	0.83 (0.69-1.00)	0.053
≥80	424	0.77 (0.64-0.94)	0.0088	≥90	202	0.69 (0.52-0.90)	0.0067
Trend		*P=0.017*		*Trend*		*P=0.0050*	
**Secondary prevention**				**Dose: 300-325mg**			
**UKTIA**				<60	117	1.18 (0.82-1.71)	0.37
<70	205	0.95 (0.72-1.26)	0.73	60-69	282	0.86 (0.68-1.09)	0.22
70-79	193	0.84 (0.62-1.12)	0.23	70-79	476	0.78 (0.65-0.94)	0.0078
≥80	128	0.72 (0.50-1.03)	0.07	80-89	275	0.82 (0.65-1.04)	0.11
**EAFT**				≥90	163	0.78 (0.57-1.07)	0.12
<70	125	0.94 (0.66-1.34)	0.74	*Trend*		*P=0.098*	
70-79	88	0.55 (0.36-0.84)	0.0054	**Dose ≥500mg**			
≥80	49	1.05 (0.60-1.85)	0.85	<60	64	1.25 (0.75-2.06)	0.39
**Subtotal**				60-69	193	0.92 (0.69-1.23)	0.58
<70	330	0.95 (0.76-1.18)	0.63	70-79	321	1.04 (0.83-1.31)	0.72
70-79	281	0.73 (0.58-0.93)	0.0094	80-89	159	0.84 (0.61-1.15)	0.28
≥80	177	0.80 (0.59-1.09)	0.16	≥90	50	0.45 (0.26-0.79)	0.005
Trend		*P=0.23*		*Trend*		*P=0.021*	

P17. Comparison of the associations between weight and the effect of aspirin versus control on the risks of all cardiovascular events and of all CV-events or death in trials in primary prevention, in secondary prevention, and both together (pooled) stratified by the dose of aspirin used. Error bars are 95%CI and size of the point estimate is proportional to the inverse of the variance.

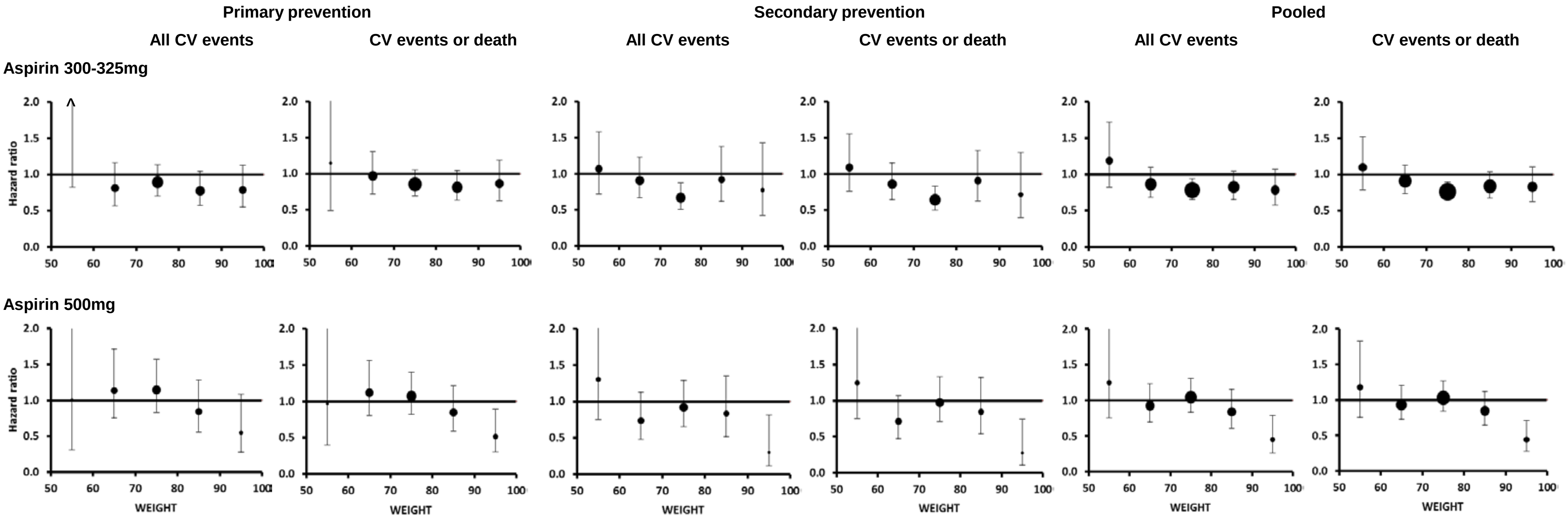

No women were included in the two primary prevention trials, but among the few women in the two secondary prevention trials, trends in weight-dependence in effect of aspirin to be appeared consistent with the overall analysis (all CV-events on 300mg vs placebo: <70kg HR=0.89, 0.63-1.25; ≥70kg 0.78, 0.48-1.28).

P18. Pooled analysis of the effect of aspirin versus control in trials in primary or secondary prevention on risks of all cardiovascular events, all CV-events or death, and major bleeding, stratified by body weight and by dose of aspirin. Error bars are 95%CI and size of the point estimate is proportional to the inverse of the variance.

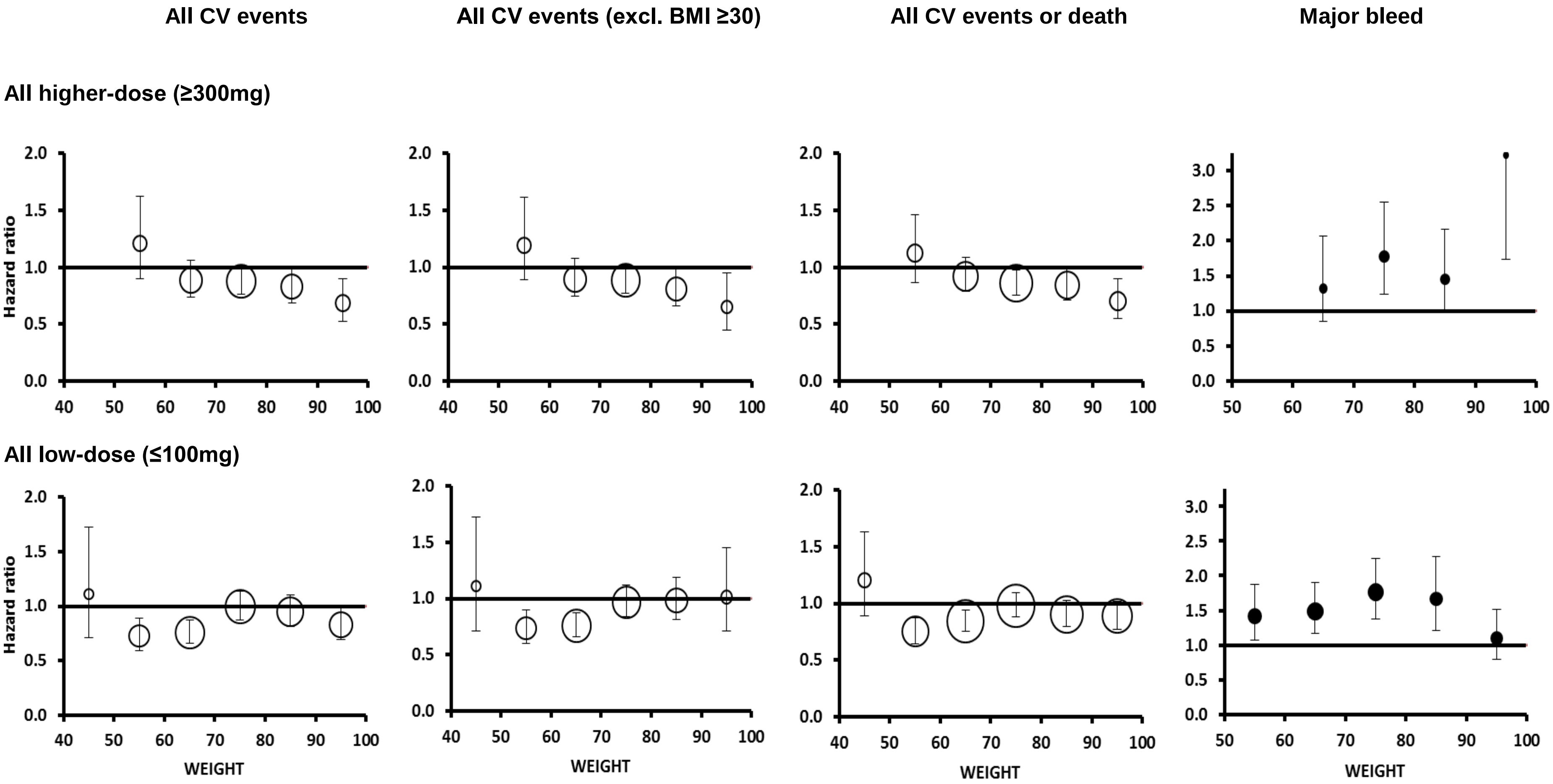

P19. A data-dependent internal validation across trials of primary prevention with aspirin versus control of the effect of aspirin when given at optimal dose for weight (75-100mg at 50-69kg; 300-325mg at 70-89kg; ≥500mg at ≥90kg) versus non-optimal dose.

Dose/weight group	Events/Patients: aspirin vs placebo<br>Outcome	HR (95% CI)	p	p (interaction)
	**All CV events**			
**Optimal**	750/23616(3.18%) vs 941/23557(3.99%)	0.76 (0.69-0.84)	<0.0001	0.0005
**Non-optimal**	1521/28711(5.30%) vs 1465/27126(5.40%)	0.95 (0.88-1.02)	0.13	
	**Stroke**			
**Optimal**	340/23616(1.44%) vs 428/23557(1.82%)	0.76 (0.66-0.88)	0.0002	0.0031
**Non-optimal**	622/28711(2.17%) vs 577/27126(2.13%)	1.01 (0.90-1.13)	0.93	
	**MI**			
**Optimal**	297/23616(1.26%) vs 406/23557(1.72%)	0.71 (0.61-0.82)	<0.0001	0.045
**Non-optimal**	630/28711(2.19%) vs 672/27126(2.48%)	0.85 (0.77-0.95)	0.0050	
	**CV death**			
**Optimal**	248/23616(1.05%) vs 297/23557(1.26%)	0.79 (0.67-0.94)	0.0062	0.0014
**Non-optimal**	653/28711(2.27%) vs 532/27126(1.96%)	1.10 (0.98-1.24)	0.10	
	**Death**			
**Optimal**	709/23616(3.00%) vs 826/23557(3.51%)	0.83 (0.75-0.92)	0.0003	0.0019
**Non-optimal**	1442/28711(5.02%) vs 1301/27126(4.80%)	1.02 (0.94-1.09)	0.70	

P20. Effect of aspirin versus control in primary of vascular events on risk of cancer and of death due to cancer during trial follow-up stratified by age and period of follow-up.

		**Weight <70kg**				**Weight ≥70kg**			
		**Any cancer**		**Cancer death**		**Any cancer**		**Cancer death**	
	**Cancers:**	**HR (95% CI)**	**p**	**HR (95% CI)**	**p**	**HR (95% CI)**	**p**	**HR (95% CI)**	**p**
**Age / period**									
All ages	***All***	1.04 (0.96-1.12)	0.36	0.90 (0.79-1.03)	0.12	1.00 (0.93-1.06)	0.89	0.98 (0.87-1.11)	0.77
	**<3**	1.18 (1.04-1.33)	0.0089	1.01 (0.81-1.25)	0.94	1.03 (0.94-1.14)	0.51	1.04 (0.87-1.25)	0.64
	**3-4.9**	1.00 (0.86-1.16)	1.00	0.94 (0.74-1.21)	0.64	0.94 (0.83-1.06)	0.32	1.01 (0.81-1.27)	0.91
	**≥5**	0.93 (0.82-1.05)	0.23	0.79 (0.63-1.00)	0.045	0.99 (0.87-1.13)	0.91	0.90 (0.72-1.12)	0.35
Age <70y	***All***	1.01 (0.93-1.10)	0.80	0.84 (0.71-1.00)	0.048	1.00 (0.93-1.08)	0.96	0.99 (0.87-1.13)	0.89
	**<3**	1.10 (0.95-1.28)	0.21	0.92 (0.67-1.27)	0.61	1.01 (0.91-1.13)	0.84	1.06 (0.85-1.30)	0.62
	**3-4.9**	0.99 (0.83-1.17)	0.89	0.77 (0.55-1.08)	0.13	0.97 (0.85-1.12)	0.69	1.09 (0.84-1.41)	0.53
	**≥5**	0.96 (0.85-1.09)	0.56	0.84 (0.66-1.08)	0.18	1.00 (0.88-1.14)	0.96	0.85 (0.68-1.07)	0.18
Age ≥70y	***All***	1.11 (0.95-1.30)	0.18	1.00 (0.81-1.24)	0.98	0.96 (0.81-1.15)	0.68	0.97 (0.75-1.25)	0.79
	**<3**	1.31 (1.07-1.61)	0.0090	1.05 (0.78-1.41)	0.74	1.09 (0.88-1.35)	0.45	0.99 (0.70-1.40)	0.96
	**3-4.9**	1.04 (0.78-1.38)	0.79	1.17 (0.82-1.68)	0.38	0.74 (0.53-1.02)	0.064	0.81 (0.50-1.31)	0.39
	**≥5**	0.62 (0.41-0.94)	0.023	0.59 (0.35-1.00)	0.051	0.86 (0.50-1.48)	0.59	1.29 (0.66-2.55)	0.46

P21. Effect of aspirin versus control (hazard ratio and 95% CI) in primary prevention on the 3-year risk of any cancer during trial follow-up stratified by weight, height, dose of aspirin and age. Error bars are 95%CI and size of the point estimate is proportional to the inverse of the variance.

Weight-stratified (p_{int} = 0.034) | **Height-stratified** (p_{int}=0.095)

All doses

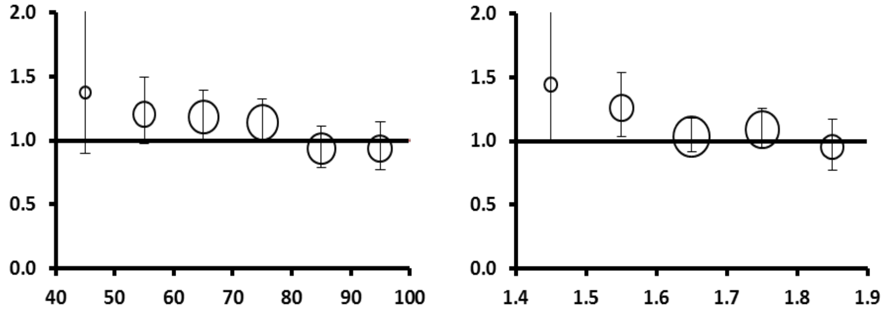

≤100mg

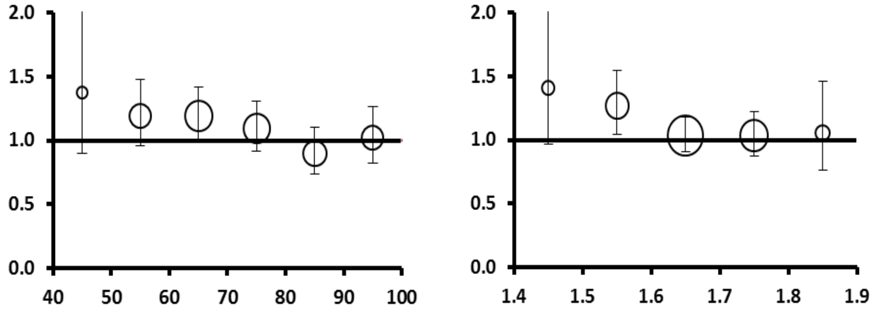

325-500mg

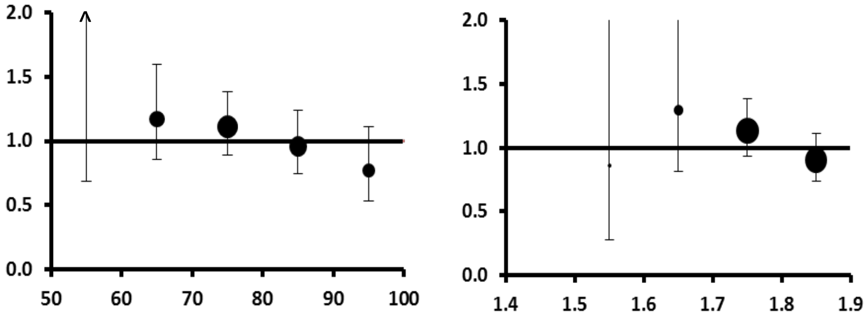

Age ≥70years

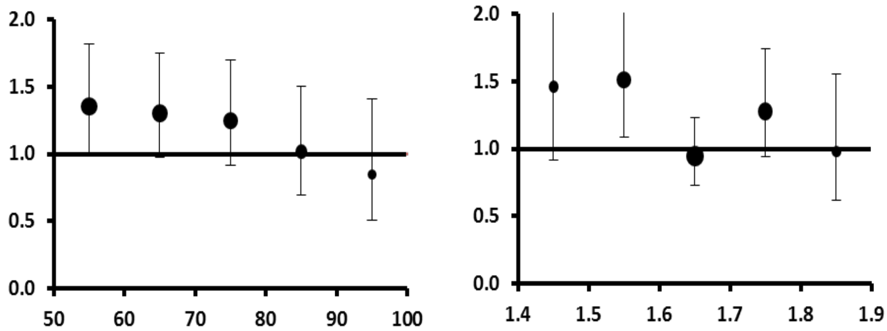

Age <70 years)

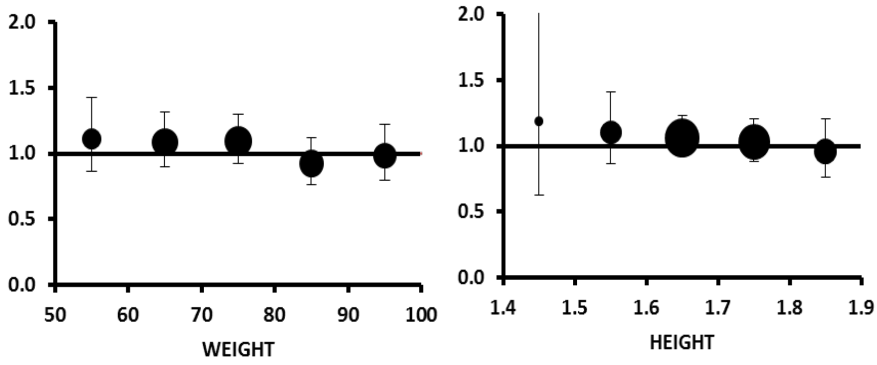

P22. Effect of aspirin versus control (hazard ratio and 95%CI) in primary prevention on risk of any cancer during trial follow-up stratified by weight, period of follow-up and dose of aspirin.
Error bars are 95%CI and size of the point estimate is proportional to the inverse of the variance.

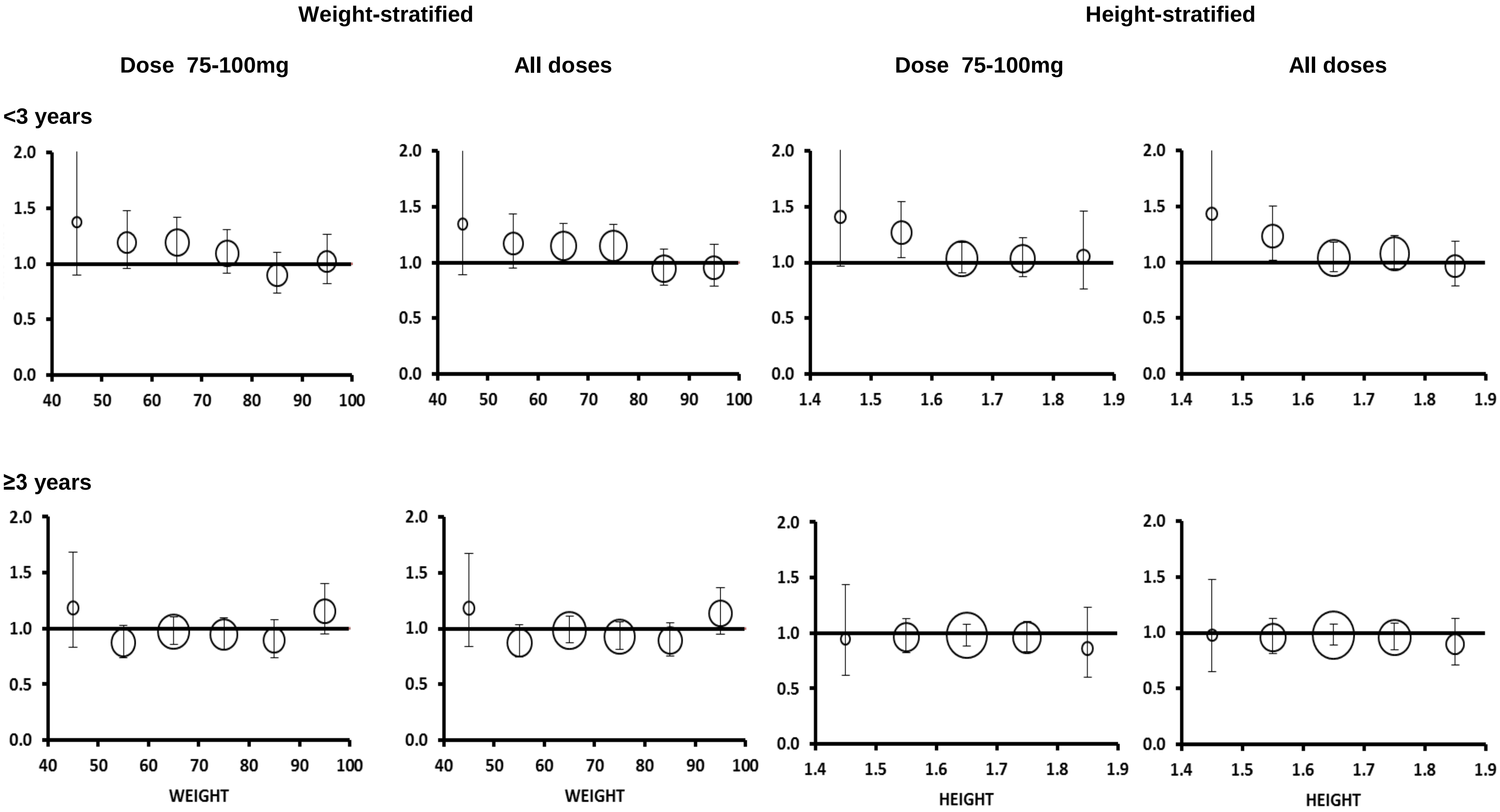

P23. Effect of aspirin versus control on in-trial cancer risk in 17,908 participants with diabetes at baseline in trials in primary or secondary prevention of vascular events.

Diabetes-associated cancers: breast; stomach; oesophagus; colorectal; uterus; pancreas, liver, kidney.

Number of cancers are greater than listed in Table 3 due to inclusion of data from trials in secondary prevention.

		**All cancers**			**Diabetes-associated cancers**	
	**Asa vs plac**	**HR (95% CI)**	**p**	**Asa vs plac**	**HR (95% CI)**	**p**
**Follow-up period**						
<3 years	181 vs 144	1.23 (0.99-1.54)	0.059	103 vs 70	1.44 (1.06-1.95)	0.019
≥3 years	161 vs 145	1.09 (0.87-1.37)	0.43	71 vs 74	0.94 (0.68-1.30)	0.72
Total	342 vs 289	1.16 (1.00-1.36)	0.057	174 vs 144	1.18 (0.95-1.48)	0.14
**Aspirin dose**						
≤100mg	300 vs 260	1.15 (0.97-1.36)	0.099	163 vs 139	1.16 (0.93-1.46)	0.19
≥300mg	42 vs 29	1.29 (0.80-2.07)	0.3	11 vs 5	1.65 (0.57-4.81)	0.36
**Sex**						
Female	177 vs 129	1.37 (1.09-1.71)	0.007	108 vs 77	1.39 (1.04-1.86)	0.028
Male	165 vs 160	1.00 (0.80-1.24)	0.99	66 vs 67	0.95 (0.67-1.33)	0.76
**Age**						
<50	28 vs 6	4.35 (1.80-10.52)	0.0011	16 vs 2	7.06 (1.62-30.77)	0.0093
50-69	188 vs 161	1.16 (0.94-1.44)	0.16	97 vs 77	1.25 (0.93-1.69)	0.14
≥70	126 vs 122	1.00 (0.78-1.28)	0.99	61 vs 65	0.91 (0.64-1.29)	0.59
**Height**						
≤1.6m	120 vs 90	1.36 (1.03-1.79)	0.028	64 vs 53	1.23 (0.85-1.76)	0.27
>1.6m	191 vs 170	1.08 (0.88-1.33)	0.45	103 vs 84	1.18 (0.88-1.57)	0.27
**Weight**						
<70kg	153 vs 113	1.34 (1.05-1.71)	0.019	83 vs 63	1.29 (0.93-1.78)	0.13
≥70kg	158 vs 147	1.06 (0.85-1.33)	0.62	84 vs 74	1.11 (0.81-1.52)	0.5
**BMI**						
<30	231 vs 202	1.11 (0.92-1.34)	0.28	120 vs 108	1.07 (0.83-1.39)	0.59
≥30	80 vs 58	1.44 (1.03-2.02)	0.035	47 vs 29	1.69 (1.06-2.68)	0.027
**Fatal events**						
**Follow-up period**						
<5 years	145 vs 124	1.16 (0.92-1.48)	0.21	60 vs 40	1.49 (1.00-2.22)	0.052
≥5 years	29 vs 41	0.71 (0.44-1.15)	0.16	12 vs 19	0.65 (0.31-1.35)	0.25
Total	174 vs 165	1.05 (0.85-1.30)	0.63	72 vs 59	1.22 (0.87-1.73)	0.25
